# Population Pharmacokinetic Modeling and Exposure‐Response Analysis for Aripiprazole Once Monthly in Subjects With Schizophrenia

**DOI:** 10.1002/cpdd.1022

**Published:** 2022-01-03

**Authors:** Xiaofeng Wang, Arash Raoufinia, Sébastien Bihorel, Julie Passarell, Suresh Mallikaarjun, Luann Phillips

**Affiliations:** ^1^ Otsuka Pharmaceutical Development & Commercialization Inc. Rockville Maryland USA; ^2^ Cognigen Corporation a *SimulationsPlus* Company Buffalo New York USA; ^3^ Fellow of the American College of Clinical Pharmacology (FCP)

**Keywords:** Abilify Maintena^®^, aripiprazole, population pharmacokinetics, schizophrenia

## Abstract

An intramuscular formulation of aripiprazole monohydrate dosed once monthly (AOM) was developed to address nonadherence with the approved oral tablets. A 3‐compartment linear population pharmacokinetic model for oral and AOM doses was developed; relative bioavailability was estimated for AOM relative to oral dosing and body mass index and sex were significant predictors of AOM absorption rate constant (longer absorption half‐life for women and absorption half‐life increases with increasing body mass index). Aripiprazole apparent oral clearance for subjects with cytochrome P450 (CYP) 2D6 poor metabolizer status and in the presence of strong CYP2D6 inhibitors was approximately half that of subjects with CYP2D6 extensive metabolizer status and 24% lower in the presence of strong CYP3A4 inhibitors. Simulations of the population pharmacokinetics were conducted to evaluate the effect of different dose initiation strategies for AOM, the effects of CYP2D6 metabolizer status, coadministration of CYP2D6 and CYP3A4 inhibitors, and missed doses. An exposure‐response model with an exponential hazard function of the model‐predicted minimum concentration (C_min_) described the time to relapse. The hazard ratio (95% confidence interval) was 4.41 (2.89‐6.75). Thus, a subject with a diagnosis of schizophrenia and C_min_ ≥ 95 ng/mL is 4.41 times less likely to relapse relative to a subject with C_min_ < 95 ng/mL.

Schizophrenia is a chronic condition that requires continual treatment to maintain symptom control and, when possible, adequate cognitive and social functioning. Nonadherence with antipsychotic medications remains a frequent risk factor associated with relapse among subjects with schizophrenia. An estimated 50% of subjects do not adhere to prescribed medication regimens for a variety of reasons.^1^ This lack of adherence leads to increased morbidity, decreased quality of life, and, consequently, an associated increase in health care costs.^2^ One of the patient‐driven reasons for nonadherence is their inability to comply with daily dosing on a long‐term basis, particularly in those who abuse or are dependent on alcohol or illegal drugs.^3^

To have a potentially positive impact on dosing adherence and patient outcomes for schizophrenia,^4^ an intramuscular formulation of aripiprazole (Abilify Maintena or aripiprazole monohydrate dosed once monthly [AOM]) administered every 4 weeks was developed, which is the first dopamine‐D_2_ receptor partial agonist available as a long‐acting injectable formulation for the treatment of schizophrenia. AOM is indicated for the treatment of schizophrenia in adult patients in the United States and Canada, and in Europe for maintenance treatment of schizophrenia in adult patients stabilized with oral aripiprazole. AOM is also approved for maintenance treatment of manic or mixed episodes of bipolar I disorder in adult patients in the United States and Canada.

Although the mechanism of action of aripiprazole remains unknown, it is believed to exert its effects through partial agonist activity at dopamine‐D_2_ and serotonin‐1A receptors, and antagonistic activity at serotonin‐2A receptors.^5^ In pivotal trials, AOM reduced the rate of, and delayed the time to, impending relapse relative to placebo or a subtherapeutic dose of aripiprazole once monthly in patients with schizophrenia.^6^, ^7^

Aripiprazole is a substrate of cytochrome P450 (CYP) 3A4 and CYP2D6 isozymes. Its activity is primarily due to the parent drug, aripiprazole, and to a lesser extent to its major metabolite, dehydro‐aripiprazole, which has been shown to have affinities for D_2_ receptors similar to the parent drug. Dehydro‐aripiprazole represents 40% of the parent drug exposure in plasma after single and multiple oral doses.^8^ The ratios of dehydro‐aripiprazole to aripiprazole pharmacokinetic (PK) parameters after the fifth monthly administration of AOM in the range of 200 mg to 400 mg were similar to that observed after oral administration (40%). Therefore, due to comparability of the ratio of aripiprazole to dehydro‐aripiprazole following AOM administration and oral administration, the population PK (popPK) analysis was performed on aripiprazole concentrations alone.

The aims of this article are to describe the popPK model developed to characterize the concentration‐time profile following oral aripiprazole dosing and AOM dosing, the popPK model–based simulations performed to help guide dosing recommendations, and the exposure‐response (E‐R) model developed to describe the time to exacerbation of psychotic symptoms/impending relapse.

## Methods

### Population PK Analysis

#### Source Data

##### Study Designs

Data from 5 clinical studies were used for the development of the popPK model for AOM and oral aripiprazole. Studies 31‐98‐206, 31‐98‐207, CN138020, and 31‐05‐244 were phase 1 studies with serial PK sampling and Study 31‐07‐246 was a phase 3 study with sparse PK sampling. Data from the AOM treatment groups from an additional phase 3 study (31‐07‐247, sparse PK sampling) were used to externally validate the popPK model. Both phase 3 studies had a 400‐mg AOM treatment arm with 1 dose reduction to 300 mg allowed. Study 31‐07‐247 also had a 50‐mg AOM treatment arm with 1 dose reduction to 25 mg allowed. The studies included treatment arms of oral aripiprazole only, AOM only, and periods with oral and AOM coadministered. The phase 3 studies also included lead‐in and stabilization stages and allowed for a dose reduction. All AOM doses were administered in the gluteus maximus with the exception of study CN138020 (13 subjects), which had doses administered to the nondominant arm or midlateral thigh. Further details of each study design are presented in Table S1.

The locally appointed ethics committee/institutional review board approved the research protocols, and voluntary written informed consent was obtained from all subjects (or their guardians) before initiation of the studies. The list of the specific study sites and institutional review boards are provided in Appendix S1.

**Figure 1:**
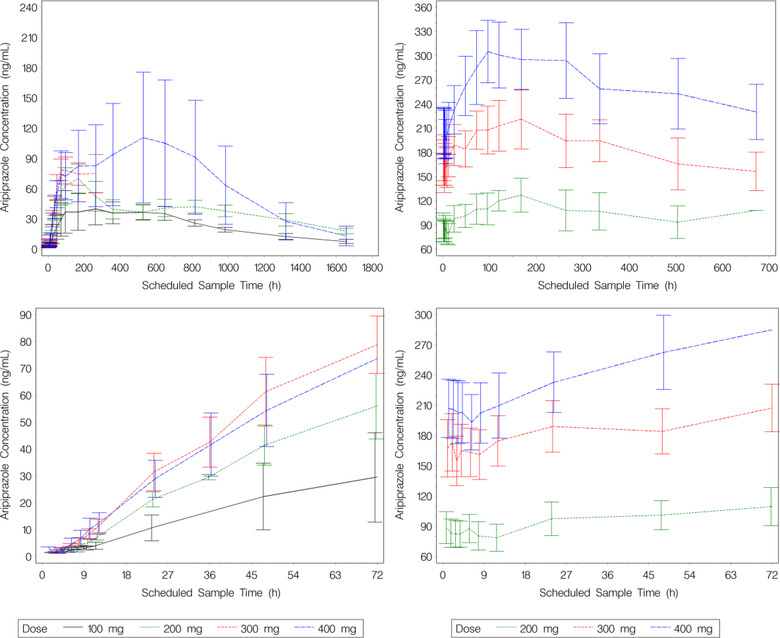
Mean ± standard error aripiprazole concentration‐time plots, stratified by AOM dose. Note: Panels in the left column display concentrations following the first injection for the full time scale (top) and truncated to the first 72 hours (bottom) (study CN138020). Panels in the right column display concentrations following the fifth injection for the full time scale (top) and truncated to the first 72 hours (bottom) (study 31‐05‐244). AOM, aripiprazole once monthly.

The overall model development population included 663 subjects with 6153 aripiprazole concentrations. There were 52 healthy subjects (oral aripiprazole only) and 611 subjects with schizophrenia or schizoaffective disorder. The external validation population (study 31‐07‐247) initially included 251 subjects with 896 aripiprazole concentrations enrolled in the 400‐/300‐mg AOM treatment arm and 121 subjects with 359 aripiprazole concentrations enrolled in the 50‐/25‐mg AOM treatment arm. The model development data set did not include doses <300 mg AOM. Initial model evaluation activities showed the final popPK model did not adequately extrapolate to describe the 50‐/25‐mg dose. Because the 50‐/25‐mg dose group was included in study 31‐07‐247 only as a low‐dose group for the noninferiority design and was not of clinical relevance, model‐predicted exposures were not calculated, and this group was excluded from the E‐R analysis (further details are provided in the Results section). The dosing and key covariate information for the model development population and for the external validation population summarized by study and overall is shown in Table S2. The summarization of the remaining covariates is provided in Table S3. The mean (standard error) concentration–time profiles for AOM following the 1st dose (study CN138020) and the mean (standard error) concentration‐time profiles for 400‐mg AOM following the fifth dose (study 31‐05‐244) are shown in Figure 1.

##### Bioanalytical Methods

Plasma samples were analyzed for determination of aripiprazole concentration using a validated high‐performance liquid chromatography with mass spectroscopy detection method. The method was linear for aripiprazole plasma concentrations between 1.00 and 250 ng/mL in studies 31‐98‐206, 31‐98‐207, and CN138020. The lower limit of quantitation was subsequently improved to 0.50 ng/mL in studies 31‐05‐244, 31‐07‐246, and 31‐07‐247. Further details are included in Appendix S2.

##### Data Analysis

All exploratory data analyses and presentations of data were performed using SAS version 9.2 (SAS Institute, Cary, North Carolina). PopPK modeling and simulation were performed using the computer program NONMEM version 6, level 2.0 (ICON plc, Dublin, Ireland); all analyses were conducted in 2011. NONMEM analyses were performed on an Intel cluster with the Linux operating system.

The first‐order conditional estimation with interaction method as implemented in NONMEM was used for all stages of model development. Model selection criteria included a statistically significant reduction of the objective function value for nested models, reductions in both residual variability (RV) and interindividual variability (IIV), improvement in the precision of parameter estimates as measured by the relative standard error (RSE), and visual examination of standard goodness‐of‐fit plots.

##### Population Pharmacokinetic Model Development

The general procedure for the development of the popPK models of oral aripiprazole and oral aripiprazole and AOM combined included examination of concentration–time plots, staged development of the base model structure, multivariable stepwise forward selection of covariates (α  =  0.05, 1 df [degree of freedom]),^9^ further evaluation of IIV and RV error models, univariate stepwise backward elimination of covariates (α  =  0.001, 1 df),^10^ evaluation of further model refinements, and model evaluation.

The development of the base model structure included 2 stages: (1) development of the base popPK model for oral aripiprazole and (2) development of the base popPK model for oral aripiprazole and AOM combined. Both models were developed using a stepwise approach sequentially introducing the data most informative for predicting a specific effect at each step. The stepwise development included the effects of CYP2D6 metabolizer status (oral data from healthy subject phase 1 studies in the absence of CYP inhibitors), administration route (data from AOM phase 1 studies added), coadministration of CYP2D6 and CYP3A4 inhibitors (oral data from healthy subject phase 1 studies in the presence of CYP inhibitors added), and sparse sampling (data from the phase 3 study added). After the first step, some subjects did not have a measured CYP2D6 metabolizer status (extensive metabolizer [EM] or poor metabolizer [PM]). Assuming that 90% of the general population had an EM status, metabolizer status was predicted for subjects with an unknown status.^9^ The prediction was performed using a mixture model, which assigns the most probable metabolism status based on the similarity of the observed concentrations for a subject to the observed data from subjects with measured EM or PM status and the percentage of EMs in the general population.

The covariates evaluated for each parameter of the oral aripiprazole and AOM combined base popPK model were as follows: first‐order absorption rate constant (K_a_) (oral): sex and body weight (WTKG); K_a_ (AOM): injection site, injection volume, needle size, sex, age, WTKG, lean body weight, and body mass index (BMI); apparent clearance (CL/F): sex, age, self‐reported race category (interpreted as per the US Food and Drug Administration), WTKG, lean body weight, BMI, presence of concomitant CYP3A4 inhibitor, liver abnormalities (all liver function laboratory values within normal range, 1 value above normal range, 2 values above normal range, etc); and apparent central volume of distribution (V_c_/F): sex, age, and WTKG. The relationships between PK parameters and continuous covariates were evaluated using linear and power functions and the relationships between PK parameters and discrete covariates were evaluated using a proportional function. A multivariable forward selection (inclusion of all statistically significant covariates when tested univariately at each step) and standard stepwise backward elimination process was used to assess the statistical significance of the evaluated covariates for inclusion into the model.

The predictability of the final model was evaluated using a simulation‐based visual predictive check (VPC) method using 1000 replicates of the model development data set. In addition, external validation was performed by applying the final popPK model to the external validation data set (study 31‐07‐247) and calculating the percent population prediction error (%PPE) and absolute value percent population prediction error (|%PPE|) (for each observation: %PPE = 100 × (observed – population predicted)/ population predicted concentration). The adequacy of the model to describe each dose group (400‐/300 mg and 50‐/25 mg) of the external validation data set was defined as the median %PPE within ±10% and the median |%PPE| <40%. The additional numerical test was performed to ensure model‐predicted exposures for this study would be appropriate for the E‐R analysis. This numerical evaluation was especially important for the 50‐/25‐mg dose because it was outside the range of doses used to develop the model.

##### Definition of Therapeutic Window

Daily oral aripiprazole doses in the range of 10 to 30 mg are considered safe and effective for the treatment of subjects with schizophrenia.^11^ Therefore, a simulation of 10 mg/day and 30 mg/day oral dosing to steady state for the 10 000 virtual EM subjects was conducted to establish the therapeutic window. The median of the simulated 10‐mg oral minimum predicted drug concentration at steady state (C_min,ss_) values (94.0 ng/mL) was used to establish the minimum of the therapeutic window as a conservative approach. Similarly, the 75th percentile of the simulated 30‐mg oral maximum predicted drug concentration at steady state (C_max,ss_) values (534 ng/mL) was selected as a conservative upper bound for the therapeutic window.

##### Population PK Model Simulations

For the purposes of examining the clinical relevance of statistically significant covariates, the individual empiric Bayesian PK parameter estimates obtained from the final popPK model were used to predict the following steady‐state exposures stratified by discrete classifications of statistically significant covariates: C_min,ss_, C_max,ss_, average drug concentration over a dosing interval, and area under the concentration‐time curve for a dosing interval (AUC_0‐τ,ss_) using numeric integration of the model derivatives within NONMEM.

Simulations were conducted to evaluate the effect of different dose initiation strategies for AOM, the effect of CYP2D6 metabolizer status, the effect of coadministration of CYP2D6 and CYP3A4 inhibitors, and the effect of missed doses on the AOM concentration‐time profile and its relationship to the oral therapeutic window. For the simulations, a population of 10 000 subjects was randomly generated. The virtual subjects were 60% men and the BMI values were log‐normally distributed on the basis of sex to be similar to the phase 3 population in the model development data set. Depending on the simulation scenario, the 10 000 subjects were assigned to a CYP2D6 EM status or a CYP2D6 PM status.

### Exposure‐Response Analysis of Time to Relapse

#### Source Data

##### Study Designs

Data from the multistage phase 3 clinical studies, studies 31‐07‐246^10^ and 31‐07‐247, as described in Table S1, were used for the development of the E‐R time to relapse model for AOM. All injections of AOM were administered in the gluteal muscle for these 2 studies.

For both studies, subjects were required to successfully complete one stage to move to the next stage of the study. For the first dose of AOM, all subjects additionally received daily oral aripiprazole for 14 days. It should also be noted that study 31‐07‐247 did not include a stabilization period for AOM and both studies also allowed 1 dose reduction during the double‐blind stage from 400 to 300 mg or from 50 to 25 mg of AOM.

The overall model development population included 615 subjects with schizophrenia, a model‐predicted aripiprazole C_min_ proximate to the event (relapse or censor), and at least 1 documented pharmacodynamic (PD) end point. A total of 120 subjects were administered oral aripiprazole, and 495 subjects were administered 400‐/300‐mg AOM. There were 121 subjects administered 50‐/25‐mg AOM excluded from the analysis because the popPK model did not adequately describe the concentrations for this dose group.

##### Definition of Time to Relapse

The E‐R model end point was defined as the time from randomization (stage 4 of study 31‐07‐246 and stage 3 of study 31‐07‐247) to exacerbation of psychotic symptoms/impending relapse. To evaluate the end point, the following assessments were collected biweekly during the randomized stage of the studies: Clinical Global Impression of Improvement (CGI‐I), CGI of Severity of Suicide (CGI‐SS), and Positive and Negative Syndrome Scale (PANSS).

Exacerbation of psychotic symptoms/impending relapse was defined as meeting any or all of the following 4 criteria during the randomization stages of the studies.

1. CGI‐I of ≥5 (minimally worse) and
2. An increase on any of the following individual PANSS items: conceptual disorganization, hallucinatory behavior, suspiciousness, unusual thought content—to a score >4 with an absolute increase of 2 on a specific item after randomization; or
3. An increase on any of the above individual PANSS items to a score >4 with an absolute increase of 4 for the sum of these PANSS items after randomization.
4. Hospitalization due to worsening of psychotic symptoms (including partial hospitalization programs), but excluding hospitalization for psychosocial reasons.
5. CGI‐SS part 1 score of 4 (severely suicidal) or 5 (attempted suicide) and/or a part 2 score of 6 (much worse) or 7 (very much worse).
6. Violent behavior resulting in clinically significant self‐injury, injury to another person, or property damage.

##### Definition of PK Exposures

Relapse events are likely dependent on exposure to lower drug concentrations proximal to the event rather than on minute‐to‐minute fluctuations in concentrations or exposure from earlier doses. Therefore, aripiprazole C_min_ at the time proximal to the event was selected as the exposure measure for the analysis.

The final PK model was applied to a data set containing the recorded dosing histories (active and placebo), date of impending relapse (PD end point), and the Bayesian PK parameter estimates to predict the defined aripiprazole C_min_ values using numerical integration in NONMEM. If the PD event occurred during the first AOM dose period, the predicted aripiprazole concentration 24 hours after the oral dose previous to the event was predicted. If the PD end point occurred during the second AOM dosing period or later, the predicted aripiprazole concentration 672 hours after the AOM dose (C_min_) and before the PD event was predicted. Concentrations were also predicted for subjects receiving placebo because the washout of previous active aripiprazole doses may not have been complete at the time of the relapse event during placebo dosing.

##### Data Analysis

All exploratory data analyses, presentations of data, and survival analyses were performed using SAS version 9.2 (SAS Institute). The PHREG procedure in SAS was used to evaluate the semiparametric Cox proportional hazards model for survival, and the LIFEREG procedure in SAS was used to assess parametric survival models, which assume the survival time follows a known distribution.

##### Exposure‐Response Model Development

Survival analysis was used to describe the E‐R relationship of time to impending relapse and aripiprazole exposure. The general procedure for model development included exploratory data analysis to determine the appropriate functional forms to evaluate for the base structural model relating the probability of survival (no event of relapse or censor) over time to C_min_ proximal to the impending relapse event or censor. Following the exploratory analysis, various functional forms of the survival models were evaluated including the semiparametric Cox proportional hazards model and parametric models including the exponential, Weibull, log‐logistic, and generalized gamma distributions. The assumption of proportional hazards was tested for the Cox proportional hazards model using the correlation between the time to event and the Schoenfeld residuals for exposure. If the Pearson correlation coefficient was statistically significant (*P* < .05), indicating that the proportional hazards assumption was not met, parametric functional forms of the survival model were assessed, which assume that the survival time follows a known distribution. These models attempt to explain the effect of drug exposure on the log of the survival function.

A univariate analysis, modeling survival time (or log of survival time) as a function of predicted aripiprazole C_min_ proximal to the impending relapse event or censor, was performed. As C_min_ was tested for inclusion in the model, a plot of the appropriate residuals (Schoenfeld or Cox‐Snell) was evaluated.

The quality of fit for the final model was evaluated using a simulation‐based VPC method using 1000 replicates of the model development data set.

## Results

### Population PK Analysis

#### Base Model Development

After completing all steps of the model development, the model that best fit the data was a 3‐compartment model with linear elimination. Zero‐order, first‐order, and sigmoid‐absorption models for oral and AOM were evaluated. Doses administered orally were best described by sigmoid absorption (infusion to depot compartment followed by first‐order absorption), which delays the time to measurable concentrations, and doses administered by injection to the arm, thigh, or gluteal muscle were best described by standard first‐order absorption.

The evaluation of the effect of CYP2D6 metabolizer status on the CL/F of aripiprazole showed it to be highly statistically significant (*P* < .000015) with the CL/F for CYP2D6 PM subjects estimated to be approximately half the CL/F of CYP2D6 EM subjects. The effect of the coadministration of CYP2D6 and CYP3A4 inhibitors on CL/F, oral K_a_, and relative bioavailability was evaluated and the presence of each inhibitor was found to significantly reduce the CL/F of aripiprazole.

With the addition of the sparse PK data from the phase 3 study (predose samples and single samples on days 7, 14, and 28 after dosing), the estimation of the relative bioavailability of the AOM dose, as compared to the oral, was assessed and the ability to estimate all of the model parameters was evaluated. Following the investigations, the final base structural model remained unchanged except for the addition of relative bioavailability and only relative bioavailability, AOM K_a_, CL/F, V_c_/F, and related IIV terms could be estimated. All other parameters were fixed to the values from the model before adding the phase 3 data. It should be noted that before the addition of the sparse PK data, all model parameters (fixed and random effects) were well estimated (%RSE: 6%‐46%), as shown in Table S4.

#### Covariate Analysis

A multivariable stepwise forward selection process was then performed. The effect of BMI as a power function and sex as a proportional function on AOM K_a_ and the effect of WTKG as a linear function on CL/F were found to be statistically significant (all individual covariate *P* values < .0037). Following the inclusion of covariates, it was determined that off‐diagonal elements of the covariance matrix (describing the covariance of IIV terms) could not be successfully estimated. Proceeding with backward elimination, the effect of WTKG on CL/F was not statistically significant (*P* > .001) and was removed from the model.

#### Final PopPK Model

**Figure 2:**
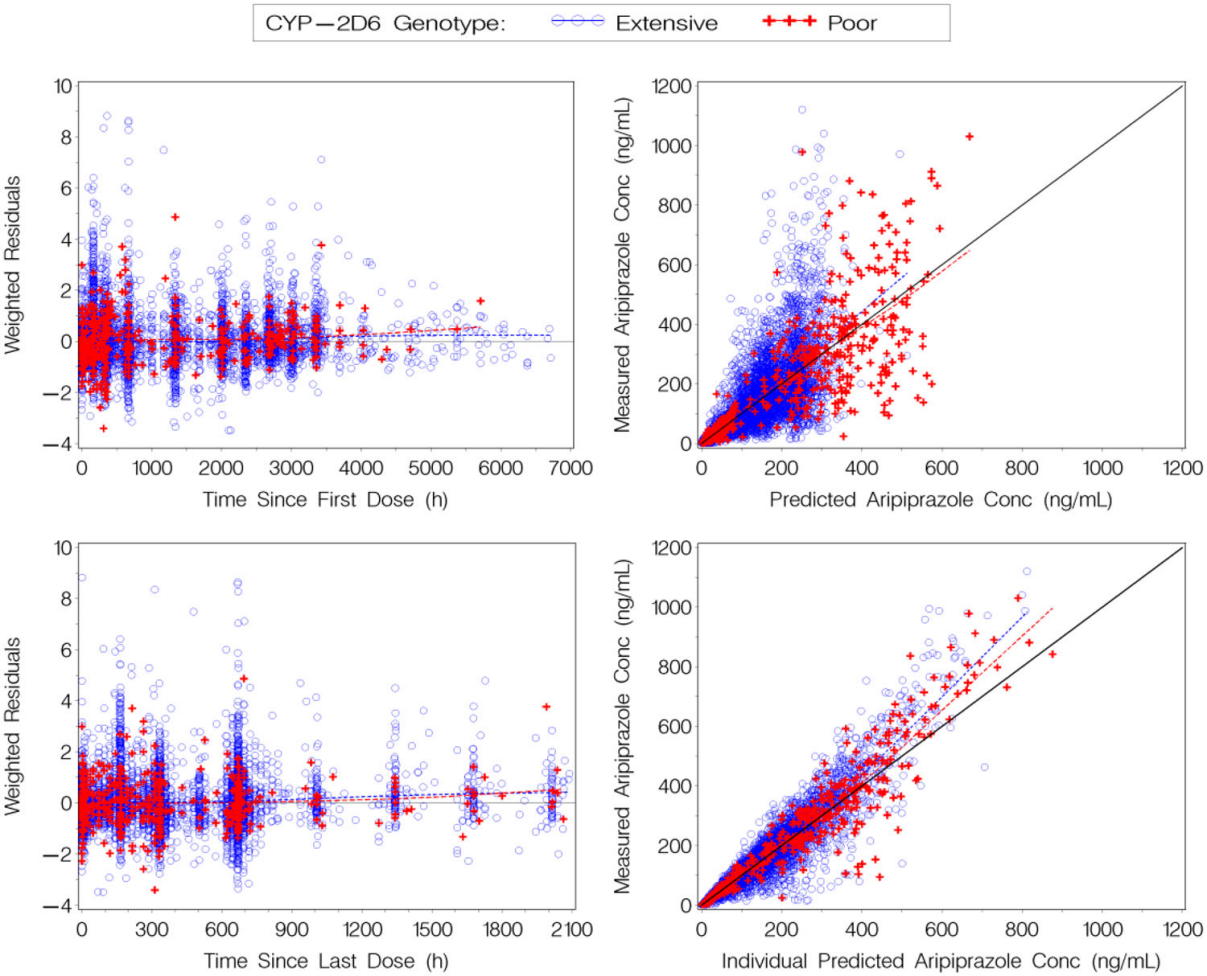
Goodness‐of‐fit plots for final model of phase 1 and phase 3 oral plus AOM data. The observed concentrations >500 ng/mL represent <1% of the data. Panels in the right column display a line of identity (solid black). All panels have loess smooth lines of the data for the extensive metabolizers (dashed blue) and for the poor metabolizers (dashed red). AOM, aripiprazole once monthly; CYP, cytochrome P450; Conc, concentration.

The final oral and AOM combined popPK model was a 3‐compartment model with sigmoid absorption for oral dosing (allowing a small lag time before absorption) and first‐order absorption for AOM dosing and included an estimated bioavailability of the AOM formulation, as compared to oral dosing. As mentioned for the base model, parameters including relative bioavailability for AOM, AOM K_a_, CL/F, V_c_/F, related IIV parameters, and RV terms were estimated in the final model, while all other parameters were fixed to values from the base model before the inclusion of the phase 3 study. The parameter estimates and associated equations of the final model are shown in Table 1, and the goodness‐of‐fit plots are shown in Figure 2.

**Table 1:** Final Model ‐ Phase 1 and Phase 3 Oral + AOM Data

	Final Parameter Estimate		Magnitude of Interindividual Variability (%CV)	
Parameter	Population Mean	%RSE	Final Estimate	%RSE
K_a_: oral first‐order absorption rate (1/h)	0.540	Fixed	65.88	Fixed
CL/F: clearance for EM (L/h)	3.71	4.0	38.34	6.9
CL/F: clearance for PM (L/h)	1.88	6.9		
CL/F: proportional change in CL/F for CYP2D6 inhibitor	–0.511	Fixed		
CL/F: proportional change in CL/F for CYP3A4 inhibitor	–0.237	Fixed		
V_c_/F: central volume (L)	93.4	8.8	124.50	15.2
Q_1_/F: intercompartmental CL/F (L/h)	0.591	Fixed	NE	NA
V_p1_/F: peripheral volume (L)	118	Fixed	NE	NA
Q_2_/F: second intercompartmental CL/F (L/h)	28.8	Fixed	NE	NA
V_p2_/F: second peripheral volume (L)	134	Fixed	NE	NA
R1: infusion rate of oral dose into depot compartment (mg/h)	9.33	Fixed	NE	NA
IM K_a_: AOM first‐order absorption rate (1/h)	0.000904	5.3	55.59	8.2
F2: relative bioavailability for AOM	1.48	4.9	NE	NA
IM K_a_: power for (BMI/28)	‐0.975	11.5	NE	NA
IM K_a_: proportional shift for men	0.346	28.9	NE	NA
Phase 1 RV (%CV)	24.23	8.4	NA	NA
Phase 3 RV (%CV)	28.11	4.7	NA	NA
Minimum value of the objective function = 48892.907				

AOM, aripiprazole once monthly; BMI, body mass index; %CV, percent coefficient of variation; CYP, cytochrome P450; EM, extensive metabolizer; IM, intramuscular; NA, not applicable; NE, not estimated; PM, poor metabolizer; %RSE, relative standard error expressed as a percent; RV, residual variability.

Note: Sigmoid absorption was described by R1, the infusion rate of the oral dose into the depot compartment, and K_a_, the oral first‐order absorption rate constant. The shrinkage of CL, V_c_, oral K_a_, and IM K_a_ was estimated as 7.3%, 52.2%, 8.2% (oral only), and 21.2% (IM only). The estimate of the clearance for extensive metabolizers was correlated with the estimate of the relative bioavailability for AOM (r  =  0.901). Related equations:

$AOM\;Ka\;=\;0.000904\;\times {\left(\frac{BMI}{28}\right)}^{-0.975}\times \left(1+0.346\times Male\right)$

$\;\frac{CL}{F}=\left(3.71\;\times EM+1.88\;\times PM\right)\;\times \left(1-0.511\times CYP2D6\right)\times \left(1-0.237\times CYP3A4\right)$

The bioavailability of AOM was estimated ≈48% higher, as compared to oral administration. The typical value of the AOM absorption half‐life (0.693/AOM K_a_) was estimated to increase with increasing values of BMI, with the AOM absorption half‐life estimated to be longer for women. For a typical subject with a BMI of 28 mg/kg^2^, the estimated AOM absorption half‐life was ≈32 days for women and 24 days for men. A graph showing the full relationship between AOM absorption half‐life and BMI for men and women is included in Figure S1. The CL/F for CYP2D6 PM subjects and CL/F in the presence of strong CYP2D6 inhibitors was approximately half that of CYP2D6 EM subjects and the CL/F in the presence of strong CYP3A4 inhibitors was ≈24% lower than that of CYP2D6 EM subjects.

The goodness‐of‐fit plots (Figure 2, right panels) exhibit some underprediction for observed concentrations > 500 ng/mL (representing <1% of the data). Given that concentrations >500 ng/mL were observed only in the phase 3 study and occurred very infrequently, this underprediction bias could be the result of inaccurate dosing records (time of previous oral dose misrecorded, an additional oral dose taken before the clinic visit and not reported, or continued oral dosing after the 14‐day lead‐in that was not reported). Thus, this bias was not considered clinically meaningful and, overall, the model described the data well. This was further supported by the uniform spread of the weighted residuals about 0 over the full duration of sampling (≈9 months) and time since previous dose up to 2100 hours (≈88 days) after dosing (Figure 2, left panels).

In addition, as shown in Table 1, all parameters were estimated with reasonable precision (RSE <30%). The magnitude of the unexplained IIV in CL/F was small (38 %CV, coefficient of variation expressed as a percent), the unexplained IIV in oral K_a_ and AOM K_a_ was moderate at 66 %CV and 56 %CV, respectively, and the unexplained IIV in V_c_/F was large (125 %CV). The rather large unexplained IIV in V_c_/F was probably related to the absorption rate‐limited PK of AOM.

#### Model Evaluation

**Figure 3:**
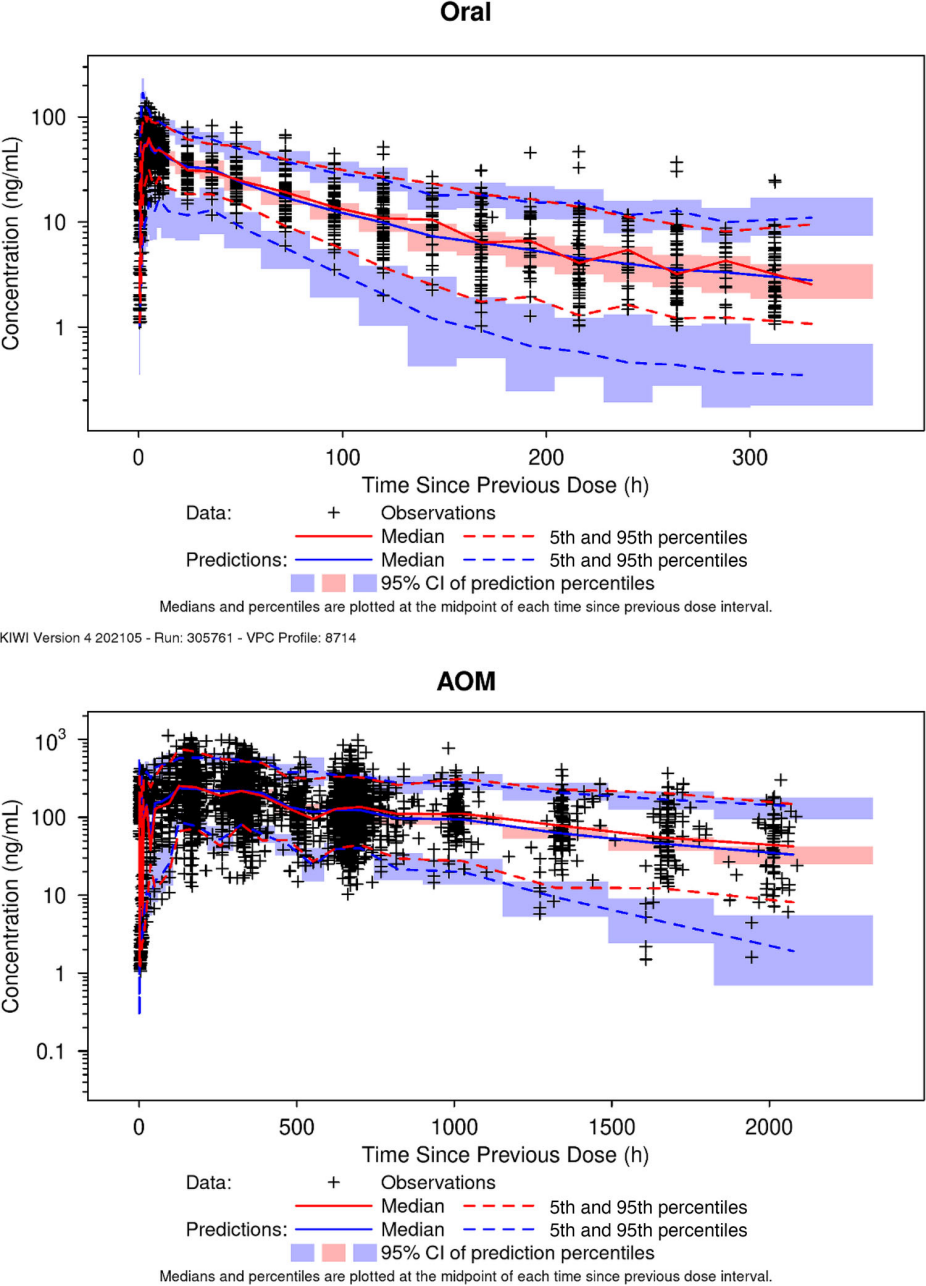
Visual predictive check of the final model, stratified by administration route. Top panel, oral. Bottom panel, AOM. AOM, aripiprazole once monthly; CI, confidence interval.

The VPC of the final popPK model stratified by route of administration for the model development data set is displayed in Figure 3. The VPC shows a close correspondence of the observed and model‐simulated 5th, median, and 95th percentiles across the full range of time since previous dose with the exception of an underprediction of the 5th percentile for oral dosing (represents ≈3 subjects). Overall, the VPC indicates that the model had no significant biases, represented an adequate fit to the data, and was appropriate for use in the simulations.

The summary statistics of the %PPE and |%PPE| for the external validation data set indicated that the final popPK model adequately described the 400‐/300‐mg dose group with a median %PPE of –6.8% and a median |%PPE| of 29.2%. However, the 50‐/25‐mg dose group did not fully meet the acceptance criteria with a median %PPE of –1.73% and a median |%PPE| of 41.3%. While the 50‐mg AOM treatment arm median |%PPE| was close to the validation criteria and only slightly above the prespecified limit of 40%, the 75th percentiles of the |%PPE| (74.6%) provided further indication that the final popPK model did not adequately extrapolate to describe the 50‐/25‐mg dose. Because the 50‐/25‐mg dose group was included in study 31‐07‐247 only as a low‐dose group for the noninferiority design and was not of clinical relevance, this treatment arm was excluded from the E‐R analyses rather than rebuild the popPK model to adequately fit concentrations following this dose. As shown in Figure S2, a VPC of the model for the validation data set was also performed and confirms the information described above.

#### Model Simulations

For the 663 subjects included in the model development data set, individual model‐predicted parameters and C_min,ss_, C_max,ss_, and AUC_0‐τ,ss_ (where τ is 24 hours for oral dosing and 672 hours for AOM dosing) were calculated using the Bayesian PK parameter estimates following virtual 10‐mg oral aripiprazole administration, and following virtual 400‐mg AOM administration. On average, the AOM absorption half‐life was ≈24 days for men and 36 days for women. The average terminal elimination half‐life was ≈7.5 days for men and women. The mean (standard deviation [SD]) CL/F for the 621 EM and 42 PM subjects was 3.90 (1.53) L/h and 2.02 (0.837) L/h, respectively. The mean (SD) CL/F for the 13 EM subjects taking CYP2D6 inhibitors and 25 EM subjects taking CYP3A4 inhibitors was 1.78 (0.665) L/h and 3.13 (1.26) L/h, respectively. For AOM dosing, the mean (SD) values of steady‐state PK exposures were as follows: C_min,ss_  =  195.9 (102.3) ng/mL, C_max,ss_  =  321.5 (126.0) ng/mL, and AUC_0‐τ_  = 178.5 (74.9) mg •  h/L.

For the 611 subjects in the model development data set who received aripiprazole AOM, there were no clinically significant changes in C_min,ss_, C_max,ss_, or AUC_0‐τ,ss_ across sex or quintiles of BMI (Figure S3). The figure further indicates that C_min,ss_ was generally higher following 400 mg of AOM dosing, as compared to 10‐mg oral aripiprazole dosing and that C_max,ss_ was generally lower following 400 mg of AOM dosing, as compared to 30‐mg oral aripiprazole dosing.

**Figure 4:**
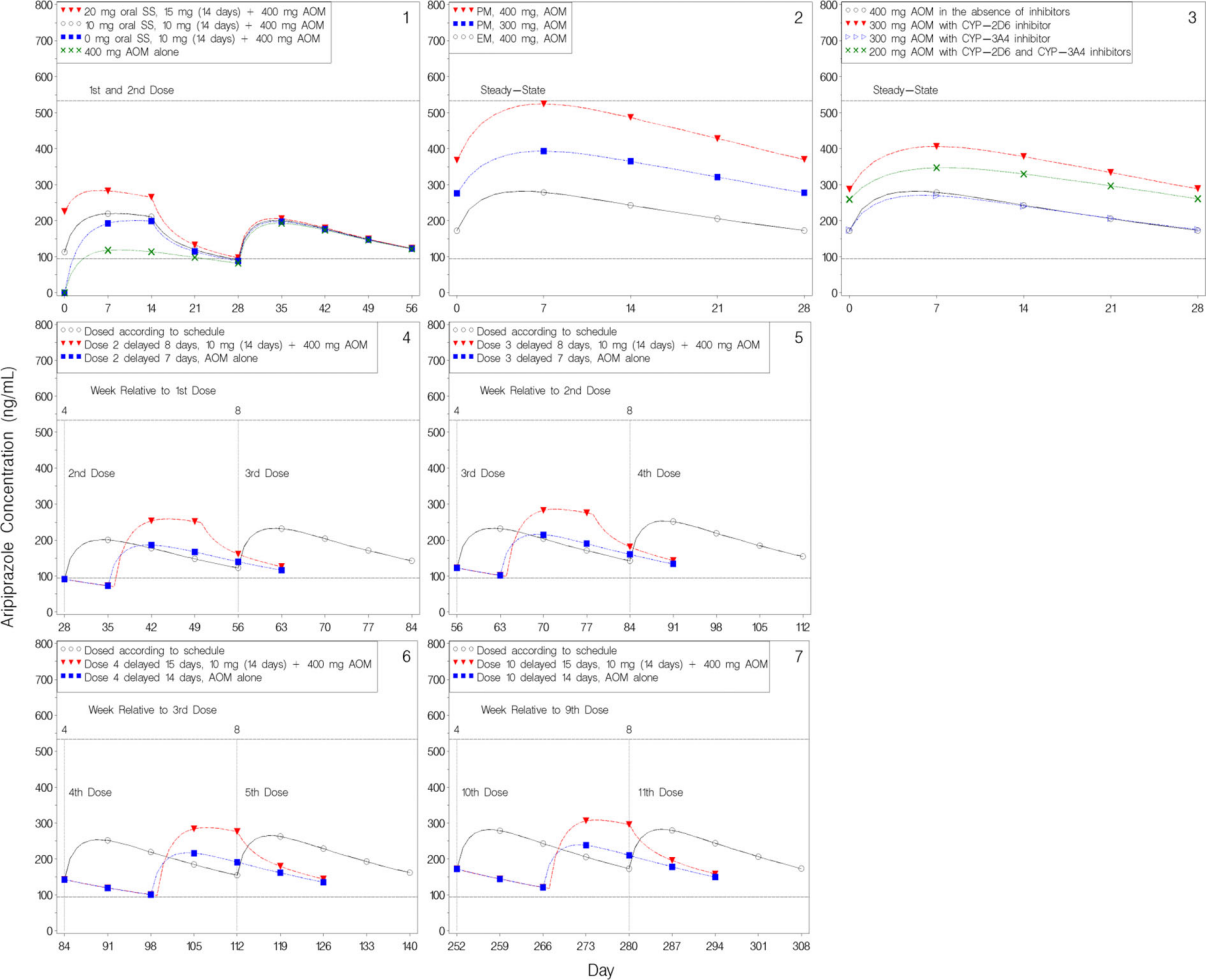
Simulated median concentration vs day. The dashed horizontal lines represent the therapeutic window. Unless specified otherwise, all groups were administered the specified amount of oral aripiprazole to steady state followed by 400‐mg AOM with or without 14 days of concomitant administration of the specified amount of oral aripiprazole. Panel 1: First 2 doses, stratified by dosing initiation scheme. Panel 2: Following steady‐state administration of specified AOM dose, concentration versus days since previous active dose for CYP2D6 poor and extensive metabolizers. Panel 3: Simulated median steady‐state concentration vs days since previous active dose. All groups were administered the specified dose of AOM to steady state with or without the presence of chronic concomitant administration of CYP2D6 or CYP3A4 inhibitors. Panels 4 and 5: Second or third dose delayed by 7 or 8 days, stratified by reinitiation of dosing without and with supplemental oral therapy, respectively. Panels 6 and 7: Fourth or 10th dose delayed by 14 or 15 days, stratified by reinitiation of dosing without and with supplemental oral therapy, respectively. AOM, aripiprazole once monthly; CYP, cytochrome P450; EM, extensive metabolizers, PM, poor metabolizers, SS, steady state.

As shown in Figure 4, panel 1, differing dose initiation schemes showed that the median concentrations were within the therapeutic window by day 7 following a 400‐mg AOM dose alone. It was also shown that with dosing of 10‐ to 20‐mg oral aripiprazole before and for 14 days after the first AOM dose (as recommended)^12^ median concentrations remained within the therapeutic window for the full time course of the first dose. As shown in Figure 4, panel 2, for subjects with a known poor CYP2D6 metabolizer status, median steady‐state concentrations after dosing of 300‐mg AOM (as recommended)^12^ remained centered within the therapeutic window, while concentrations following dosing of 400‐mg AOM approached the upper limit of the therapeutic window. As shown in Figure 4, panel 3, a dose reduction to 300‐mg aripiprazole AOM for chronic coadministration of CYP2D6 or CYP3A4 inhibitors^12^ and a dose reduction to 200‐mg aripiprazole AOM for chronic coadministration of both inhibitors^12^ (recommended dose adjustments based on simulations) is expected to result in median steady‐state concentrations remaining within the therapeutic window.

To further investigate the repercussions of delayed dosing, simulations delaying the second, third, fourth, and 10th aripiprazole AOM doses by differing lengths of time were conducted; the effect of reinitiating AOM dosing with or without concomitant oral therapy was also assessed. The median aripiprazole plasma concentration–time profiles for these simulations are displayed in Figure 4, panels 4 through 7. When the second and third doses of aripiprazole AOM were delayed by more than 1 week, the median aripiprazole concentrations for the AOM dose administered with 14 days of concomitant oral therapy reached levels similar to the doses administered on schedule by approximately the third day after dosing. After the third day, the concentrations declined and returned to a concentration pattern similar to that of reinitiating dosing following a 1‐week delay without oral therapy. Similar patterns were observed when the fourth and 10th doses were delayed by >2 weeks followed by reinitiation with oral concomitant therapy, as compared to a 2‐week delay followed by AOM dosing alone.

### Exposure‐Response Analysis of Time to Relapse

The analysis data set included 85 impending relapse events (48 events or 56.5% for placebo and 37 events or 43.5% for the 400‐/300‐mg AOM dose group). The analysis data set included 530 censored subjects (72 subjects or 13.6% for placebo and 458 subjects or 86.4% for the 400‐/300‐mg AOM dose group). As a result of subjects receiving aripiprazole before randomization to placebo, the mean (SD) predicted aripiprazole C_min_ was 47.8 (65.3) ng/mL for subjects assigned to placebo and 180.9 (83.1) ng/mL for subjects assigned to 400‐mg AOM. Furthermore, the mean (SD) model‐predicted aripiprazole C_min_ for all dose groups (placebo, 300‐mg AOM, and 400‐mg AOM) was 101.7 (90.0) ng/mL in impending relapse subjects and 161.5 (93.6) ng/mL in censored subjects.

The results from the dose‐response analysis showed that the time to impending relapse was significantly shorter for subjects in the placebo group as compared with subjects in the aripiprazole AOM 400‐/300‐mg group (hazard ratio, 8.009; *P* < .0001; log rank test).

**Figure 5:**
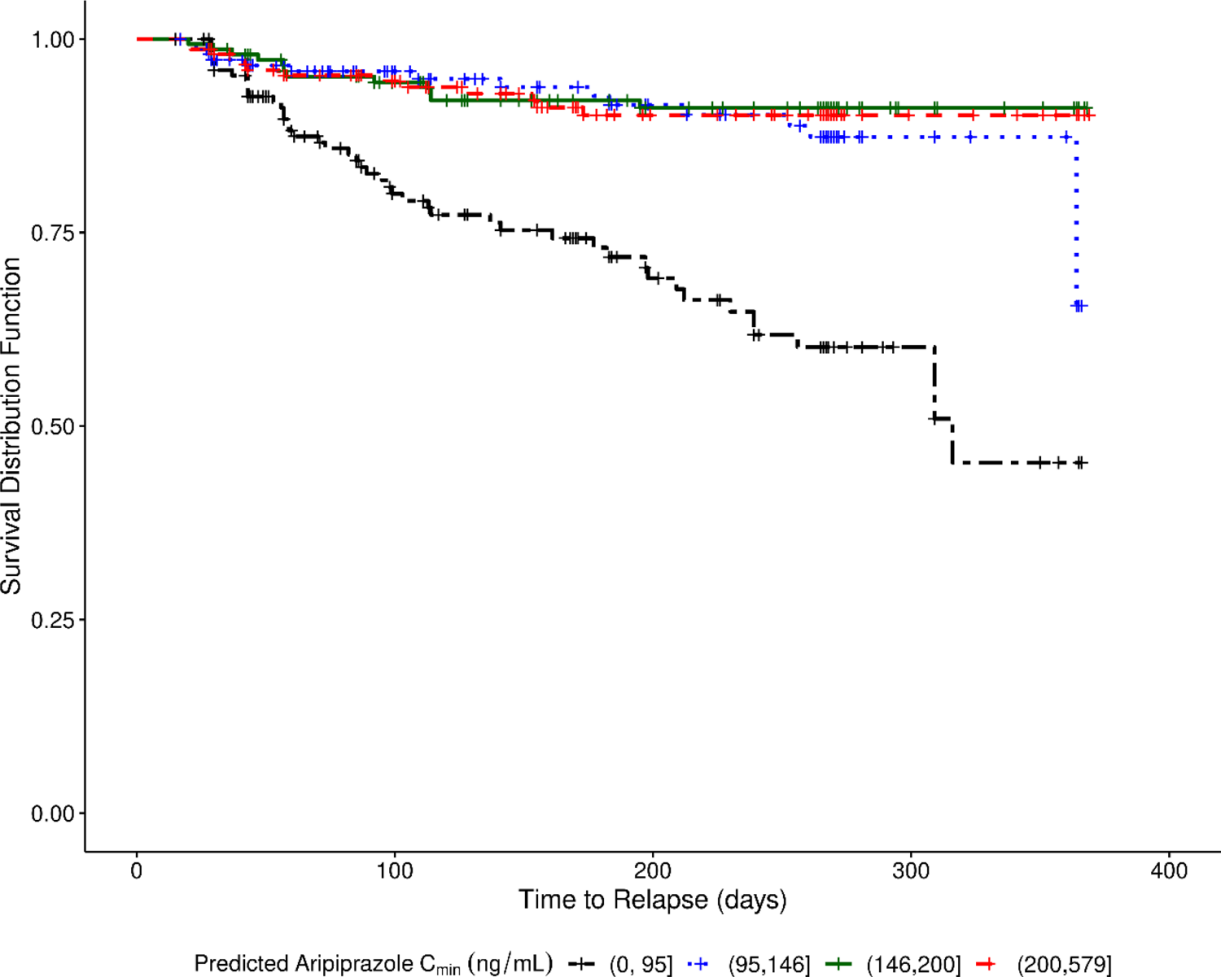
Kaplan‐Meier plot of survival (no relapse) vs time for placebo and 400‐/300‐mg aripiprazole AOM treatment groups, grouped by quartiles of predicted aripiprazole C_min_. Quartile 1 is C_min_ ≤95 ng/mL; Quartile 2 is 95 < C_min_ ≤146 ng/mL; Quartile 3 is 146 <C_min_ ≤200 ng/mL; and Quartile 4 is 200 <C_min_. Note: The minimum model‐predicted C_min_ was not 0 for the placebo treatment arm because of incomplete washout of prior oral dosing. The minimum model‐predicted C_min_ was 0.000329 ng/mL. AOM, aripiprazole once monthly; C_min_, minimum predicted drug concentration.

The Kaplan‐Meier plot of time to impending relapse, stratified by quartiles of the model‐predicted aripiprazole C_min_ proximate to the time of relapse or censoring, is shown in Figure 5. For C_min_ > 95 ng/mL (the highest 3 quartiles), there was little difference in the survival distribution function regardless of concentration.

Initial modeling indicated that the proportional hazard assumption was not met. Thus, parametric functional forms of the survival model were assessed. The exponential distribution resulted in the best fit to the data. However, there was bias in the model predictions for C_min_ concentrations >95 ng/mL (the highest 3 quartiles). The model predicted a decreasing E‐R relationship between C_min_ and time to relapse in the 3 highest quartiles, whereas the observed data showed a similar time to relapse in the 3 highest concentration quartiles. The model misfit at the highest predicted concentrations may be the result of unknown influences, or it could be indicative of a concentration‐threshold relationship of efficacy instead of a continuous relationship.

**Figure 6:**
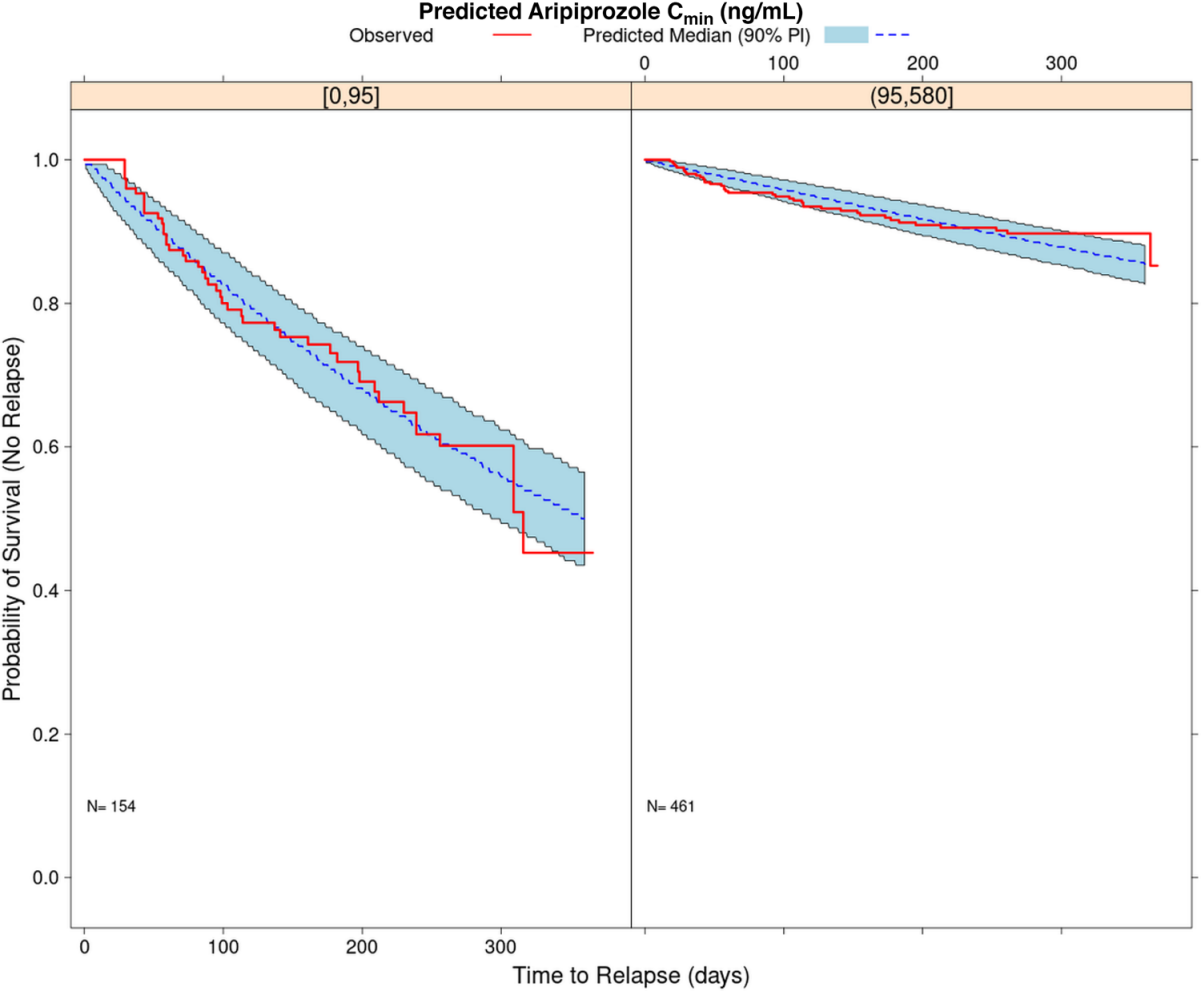
Observed (red line) and model‐predicted (blue dashed line) probability of survival vs time for groups of predicted aripiprazole 0 < C_min_ ≤95 ng/mL and 95 < C_min_ ≤580 ng/mL. The shaded regions show the 90% prediction interval of the model‐predicted probability of survival vs time. C_min_, minimum predicted drug concentration; PI, prediction interval.

To explore the potential of a concentration‐threshold relationship for efficacy, 2 groups of predicted C_min_ were evaluated (<95 ng/mL vs ≥95 ng/mL). A statistically significant relationship between time to impending relapse and grouped aripiprazole C_min_ was detected (Table 2). The hazard ratio for aripiprazole C_min_ ≥95 ng/mL to that for aripiprazole C_min_ <95 ng/mL was equal to 4.41 with a 95% confidence interval ranging from 2.89 to 6.75. Thus, a subject with a diagnosis of schizophrenia and a predicted aripiprazole C_min_ ≥95 ng/mL is 4.41 times less likely to relapse, as compared to a subject with a predicted C_min_ <95 ng/mL. As illustrated in Figure 6, there was good agreement between the observed and model‐predicted estimates of the survival distribution function for both groups of model‐predicted C_min_, illustrating a threshold concentration effect whereby predicted concentrations ≥95 ng/mL are associated with greater efficacy over time.

**Table 2:** Parameter Estimates for Exponential Survival Model of Impending Relapse as a Threshold Function of Model‐Predicted Aripiprazole C_min_ (<95 ng/mL and ≥95 ng/mL)

Parameter	Parameter Estimate	Standard Error	95% Confidence Interval		*P* Value
Intercept	6.256	0.1474	5.97	6.55	<.0001
Predicted aripiprazole (C_min_ ≥ 95 ng/mL) proximate to the event	1.484	0.2177	1.06	1.91	<.0001
Calculated hazard ratio of expected survival time for C_min_ ≥95 ng/mL:C_min_ <95 ng/mL	4.41	NA	2.89	6.75	NA

C_min_, model‐predicted minimum aripiprazole concentration; NA, not applicable.

Note: Calculated ratio of expected survival time was calculated as *e* ^parameter estimate^.

## Discussion

### Population PK Analysis

This publication describes the popPK analysis of oral‐ and AOM‐administered aripiprazole in healthy subjects or subjects with schizophrenia or schizoaffective disorder. The studies in this analysis included phase 1 trials designed to evaluate the effect of CYP2D6 and CYP3A4 inhibitors and the effect of CYP2D6 polymorphism on the PK of orally administered aripiprazole. The phase 3 study (study 31‐07‐246) also measured the CYP2D6 genotype in almost all subjects, thus extending the evaluation of its effect on the PK of AOM aripiprazole.

The analysis showed that a linear 3‐compartment model with sigmoid absorption for oral formulation and first‐order absorption for the AOM formulation was found to best characterize aripiprazole concentrations. The sigmoid absorption indicated that the amount of drug absorbed per unit of time increased, which created a short lag time followed by a steeper ascent in the amount of drug absorbed, whereas, for AOM, the amount of drug absorbed per unit of time declined at a constant exponential rate.

The estimated model parameters showed the elimination of the AOM formulation to be absorption‐rate limited with an average half‐life of absorption of 28 days and an average terminal elimination half‐life of 7.5 days. The covariate analysis further showed that the absorption rate of the AOM dose was dependent on BMI and sex.

However, the model‐predicted C_min,ss_, C_max,ss_, and AUC_0‐τ,ss_ parameters for the popPK analysis population (BMI ranging from 15 to 61 kg/m^2^) did not exhibit any trends with respect to BMI or sex. Concomitant administration of CYP2D6 inhibitors or CYP3A4 inhibitors and CYP2D6 metabolizer status were determined to have a statistically significant effect on clearance. In a popPK analysis published by Koue T et al., the mean half‐life of oral aripiprazole was reported to be 44.2 and 76.3 hours in EMs and PMs, respectively, and 67.2 and 77.9 hours for subjects without and with coadministration of itraconazole.^13^ This represented an ≈42% reduction in clearance for CYP2D6 PMs and a 14% reduction in CL/F related to the coadministration of CYP3A4 inhibitors.^13^ These results, obtained using independent data, are consistent with the effects of the CYP2D6 poor metabolism and CYP3A4 inhibition predicted in the current popPK model.

Based on previously established safe and efficacious doses of oral aripiprazole (10 mg/day and 30 mg/day),^11^ the model was used to simulate the associated concentration range to establish a therapeutic window of 94.0 ng/mL to 534 ng/mL. Simulations were also conducted to evaluate the effect of different dose initiation strategies for aripiprazole AOM, effect of CYP2D6 metabolizer status, effect of coadministration of CYP2D6 and CYP3A4 inhibitors, and effect of missed doses on the AOM PK profile and its position relative to the therapeutic window.

In general, the simulations demonstrated that the use of 400‐mg AOM is an appropriate regimen for subjects requiring antipsychotic therapy. The recommended initiation regimen of 400‐mg AOM plus concomitant use of 10‐ to 20‐mg oral therapy per day for 14 days enables subjects to achieve and maintain therapeutic concentrations over a 28‐day period.^12^ Additionally, simulations indicated that the following dose adjustments are anticipated to achieve and maintain therapeutic concentrations over a 28‐day period:

- 300‐mg AOM for subjects with a CYP2D6 PM status^12^; and
- 300‐mg AOM for subjects with chronic coadministration of CYP2D6 or CYP3A4 inhibitors^12^; and
- 200‐mg AOM for subjects with chronic coadministration of both CYP inhibitors.^12^

The recommended dosing interval for AOM is every 28 days; however, based on simulations, an AOM dose can be delayed by up to 1 week during the early initiation of therapy (eg, after the second or third dose) or delayed for up to 2 weeks during maintenance therapy (eg, the fourth and subsequent doses) without requiring concomitant oral therapy upon reinitiation of AOM therapy.^12^ If subjects delay their dose for >1 week during initiation or >2 weeks during maintenance, the simulations demonstrated that reinitiation of AOM dosing with concomitant administration of oral therapy for 14 days achieves therapeutic concentrations and maintains them for the remainder of the subsequent 28‐day dosing interval.^12^

### Exposure‐Response Analysis of Time to Relapse

The impending time to relapse data from Studies 31‐07‐246 and 31‐07‐247 provided a valuable source of information to assess whether the efficacy of the AOM formulation is consistent with the therapeutic window based upon daily oral dosing of aripiprazole of 10 to 30 mg. The time‐to‐event E‐R analysis using the exponential survival model suggested that the risk of impending relapse increases as the model‐predicted aripiprazole C_min_ decreases below 95 ng/mL. Furthermore, a subject with a diagnosis of schizophrenia and a predicted concentration ≥95 ng/mL is 4.41 times less likely to relapse, as compared to a subject with a predicted concentration <95 ng/mL, which is consistent with the oral therapeutic window and supports a minimally effective C_min_ of 95 ng/mL for the prevention of impending relapse with the use of the AOM formulation. With the findings of the popPK model and the time to impending relapse analyses, the therapeutic window can be used to bridge efficacy from gluteal site administration of AOM to a different muscle (eg, deltoid). Provided that C_min,ss_ following administration at alternative sites remains >95 ng/mL, efficacy is not expected to be compromised.

## Conclusions

A popPK model describing concentrations following oral and AOM administration was developed showing that the AOM formulation exhibits absorption rate‐limited PK and is 48% more bioavailable than the oral formulation. The model also showed elimination was significantly decreased by co‐administration of CYP2D6 and CYP3A4 inhibitors and was ≈50% slower for subjects classified as CYP2D6 PMs.

Simulations of the popPK model, following various AOM dosing regimens (including oral initiation and coadministration of CYP2D6 or CYP3A4 inhibitors) for subjects classified with poor or extensive CYP2D6 metabolism, allowed for guidance of dosing recommendations.

E‐R analyses showed a subject with a diagnosis of schizophrenia and a predicted C_min_ ≥95 ng/mL is ≈4.41 times less likely to relapse, as compared to a subject with a predicted C_min_ < 95 ng/mL. This threshold concentration was equivalent to the median model‐predicted C_min,ss_ for subjects administered 10‐mg oral aripiprazole, which has also been shown to be efficacious for adult subjects with a diagnosis of schizophrenia. Therefore, it is expected that AOM administration to alternative sites that maintain concentrations above this threshold should not compromise efficacy.

## Conflicts of Interest

Cognigen Corporation received financial support from Otsuka Pharmaceutical Development & Commercialization, Inc. to perform these analyses. Sébastien Bihorel, Julie Passarell, and Luann Phillips are employees of Cognigen Corporation and have shares/stock options in Simulations Plus, Inc. Xiaofeng Wang, Arash Raoufinia, and Suresh Mallikaarjun are employees of Otsuka Pharmaceutical Development & Commercialization, Inc.

## Funding

These studies were funded by Otsuka Pharmaceutical Development & Commercialization, Inc., Rockville, Maryland. The population pharmacokinetic and exposure‐response analyses reported above were funded by Otsuka Pharmaceutical Development & Commercialization, Inc., Rockville, Maryland.

## Author Contributions

Luann Phillips and Arash Raoufinia were involved in the design of the data analysis plan, development of the population pharmacokinetic model, performance of simulations, and interpretation of the results. Sébastien Bihorel was involved in the development of the population pharmacokinetic model, performance of simulations, and interpretation of the results. Julie Passarell was involved in the design of the data analysis plan, execution of the exposure‐response analyses, and interpretation of the results. Suresh Mallikaarjun was involved in the design of the data analysis plan and interpretation of the results. Xiaofeng Wang was involved with the interpretation of data in the application of the analyses. All authors participated in the preparation and review of the article. All authors read and approved the final version of the article.

## Supporting information

Supporting material
