# Supplementary material for: Population Pharmacokinetic Modeling and Exposure‐Response Analysis for Aripiprazole Once Monthly in Subjects With Schizophrenia
Source: Clin Pharmacol Drug Dev. 2022 Jan 3;11(2):150–64. doi: 10.1002/cpdd.1022 (PMC10026531; doi:10.1002/cpdd.1022)
Supplement: Supplementary file 1 — Supporting material [file CPDD-11-150-s001.docx]

| Table S1. Summary of Study Designs | | | | |
| --- | --- | --- | --- | --- |
| Study Number (phase) Reference | Study Design Features | Subject Description | Dosing | Pharmacokinetic Sample Times |
| 31-98-206 (phase 1) Reference: 1 | Double-blind cross-over Placebo-controlled DDI: Ketoconazole | Healthy | - Single dose oral - Days 1 and 16 - 15 mg aripiprazole - 200 mg qd ketoconazole Days 15-28 | Day 1 and Day 16: Predose and 0.5, 1, 2, 3, 4, 5, 6, 8, 10, 12, 24, 36, 48, 72, 96, 120, 168, 216, and 312 hours postdose |
| 31-98-207 (phase 1) Reference: 2 | Open-label parallel CYP2D6 EM CYP2D6 PM DDI: Quinidine EM | Healthy | - Single dose oral - 10 mg aripiprazole - 166 mg quinidine qd Days 1-13 | Day 1: Predose and 1, 2, 3, 4, 6, 8, 12, 24, 48, 72, 96, 120, 144, 168, 192, 216, 240, 264, 288, and 312 hours postdose |
| CN138020 (phase 1) Reference: 3 | Open-label, 2-phases non-randomized sequential ascending dose | Schizophrenia or schizoaffective disorder | Phase 1:   - Single dose standard intramuscular - 5 mg aripiprazole   Phase 2:   - Single dose AOM - 15, 50, and 100 mg non-dominant arm 200, 300, or 400 mg mid-lateral thigh | Day 1: Predose and 0.25, 0.5, 1, 2, 3, 4, 6, 8, 10, 12, 24, 36, 48, 72, 96, 120, 168, 264, 360, 456, and 528 hours postdose  For doses > 200 mg: Days 28, 35, 42, 56, 70, 84, 112, 140, and 168 hours postdose, or until 2 consecutive LLOQ |
| 31-05-244^a^ (phase 1b) Reference: 4 | Open-label parallel | Schizophrenia | - Stabilized on 10 mg qd oral for 14 days prior to first injection - Continued on 10 mg qd oral for 14 days post first injection - Multiple dose AOM 5 months - 200, 300, or 400 mg q4weeks | Injections 1 to 4 (Days 1, 28, 56, and 112): Predose, Day 7, Day 14, and Day 21 for each injection  Injection 5:  Predose and 1, 2, 3, 4, 6, 8, 12, 24, 48, 72, 96, 120, 168, 264, 336, 504, and 672 hours postdose |
| 31-07-246 (phase 3) Reference: 5 | Double-blind, 4 phases Placebo-controlled randomized  Stage 1: Convert to oral aripiprazole  Stage 2: Stabilize dose of oral aripiprazole  Stage 3: Stabilize dose of AOM (3 doses q4weeks)  Stage 4: Double-blind period | Schizophrenia | - Stages 1 and 2: Stabilized on 10-20 mg qd oral prior to first injection - Stage 3:  Continued on 10-15 mg qd oral for 14 days post first injection 400 mg AOM q4weeks Single decrease to 300 mg allowed for tolerability  Return to 400 mg if needed Total of 3 AOM doses - Stage 4: 400 or 300 mg AOM 1 year Single dose modification allowed (see above) | Study Stage 3: Injection 1: Predose, 7 days, 14 days, and 28 days  Study Stage 3 Injections 2, 3, and 4: 28 days  Study Stage 4 Injections 1 and 2: 14 days and 28 days |
| 31-07-247^b^ (phase 3) Reference: 6 | Non-inferiority, double-blind, 3 phases Placebo-controlled randomized  Stage 1: Convert to oral aripiprazole  Stage 2: Stabilize dose of oral aripiprazole  Stage 3: Double-blind period | Schizophrenia | - Stages 1 and 2: Stabilized on 10-30 mg qd oral prior to first injection - Stage 3: Randomized to continuation of 10-30 mg qd oral with 400 mg AOM or 50 mg AOM - AOM treatments: Continued on 10-20 mg qd oral for 14 days post first injection - Single dose reduction of AOM from 400 mg to 300 mg or from 50 mg to 25 mg - Return to higher dose (if needed) | Stage 3 Injections 1-5: predose (Day 28 of each injection) |
| AOM, aripiprazole once monthly; CYP, cytochrome P450; DDI, drug-drug interaction; EM, extensive metabolizer; LLOQ, lower limit of quantitation; PM, poor metabolizer; q4weeks, once every 4 weeks; qd, once daily.  ^a^ Additional samples collected more than 672 hours postdose of the fifth injection were not included in the pharmacokinetic analysis because subjects could resume dosing of oral aripiprazole or other antipsychotic medications during this time period and the amount and administration times of these doses were not available.  ^b^ Since the 50 mg/25 mg AOM treatment arm was included in the trial as a sub-therapeutic dose of aripiprazole to test assay sensitivity and was not a clinically relevant dose, data from this arm was excluded from the population PK analysis. Because the focus of the analysis was aripiprazole AOM, the dose group receiving oral aripiprazole only was also excluded. | | | | |

| Table S2. Summary Statistics of Dosing and Key Covariates | | | | | | | | |
| --- | --- | --- | --- | --- | --- | --- | --- | --- |
| Subject Characteristic | Description | Study  31-98-207 (DDI CYP2D6) N = 29 Reference: 1 | Study  31-98-206 (DDI CYP3A4) N = 23 Reference: 2 | Study  CN138020 (AOM Single Dose) N = 13 Reference: 3 | Study  31-05-244 (AOM Multiple Dose) N = 37 Reference: 4 | Study  31-07-246 (Phase 3) N = 561 Reference: 5 | Overall N = 663 | Study  31-07-247 N = 251^a^ Reference: 6 |
| Categorical | | | | | | | | |
| Sex, n (%) | Male | 14 (48.3) | 18 (78.3) | 11 (84.6) | 37 (100.0) | 339 (60.4) | 419 (63.2) | 151 (60.2) |
|  | Female | 15 (51.7) | 5 (21.7) | 2 (15.4) | 0 (0.0) | 222 (39.6) | 244 (36.8) | 100 (39.8) |
| Race, n (%) | White/Caucasian | 20 (69.0) | 12 (52.2) | 3 (23.1) | 12 (32.4) | 322 (57.4) | 369 (55.7) | 154 (61.4) |
|  | Black/African American | 2 (6.9) | 2 (8.7) | 8 (61.5) | 21 (56.8) | 109 (19.4) | 142 (21.4) | 50 (19.9) |
|  | American Indian/Alaskan Native | 0 (0.0) | 0 (0.0) | 0 (0.0) | 0 (0.0) | 2 (0.4) | 2 (0.3) | 0 (0.0) |
|  | Asian | 0 (0.0) | 1 (4.3) | 0 (0.0) | 1 (2.7) | 102 (18.2) | 104 (15.7) | 27 (10.8) |
|  | Native Hawaiian or other Pacific Islander | 0 (0.0) | 0 (0.0) | 0 (0.0) | 0 (0.0) | 1 (0.2) | 1 (0.2) | 0 (0.0) |
|  | Other/Unknown | 7 (24.1) | 8 (34.8) | 2 (15.4) | 3 (8.1) | 25 (4.5) | 45 (6.8) | 20 (8.0) |
| Number of Liver Function Abnormalities, n (%) | 0 | 27 (93.1) | 20 (87.0) | 10 (76.9) | 33 (89.2) | 435 (77.5) | 525 (79.2) | 198 (78.9) |
|  | 1 | 2 (6.9) | 3 (13.0) | 2 (15.4) | 3 (8.1) | 93 (16.6) | 103 (15.5) | 34 (13.5) |
|  | 2 | 0 (0.0) | 0 (0.0) | 1 (7.7) | 1 (2.7) | 27 (4.8) | 29 (4.4) | 14 (5.6) |
|  | 3 | 0 (0.0) | 0 (0.0) | 0 (0.0) | 0 (0.0) | 6 (1.1) | 6 (0.9) | 4 (1.6) |
|  | 4 | 0 (0.0) | 0 (0.0) | 0 (0.0) | 0 (0.0) | 0 (0.0) | 0 (0.0) | 1 (0.4) |
| Oral Dose (mg), n (%) | 10 | 29 (100.0) | 0 (0.0) | 0 (0.0) | 37 (100.0) | 355 (63.3) | 421 (63.5) | 155 (61.8) |
|  | 15 | 0 (0.0) | 23 (100.0) | 0 (0.0) | 0 (0.0) | 193 (34.4) | 216 (32.6) | 96 (38.2) |
|  | 20 | 0 (0.0) | 0 (0.0) | 0 (0.0) | 0 (0.0) | 8 (1.4) | 8 (1.2) | 0 (0.0) |
|  | 30 | 0 (0.0) | 0 (0.0) | 0 (0.0) | 0 (0.0) | 4 (0.7) | 4 (0.6) | 0 (0.0) |
|  | AOM only | 0 (0.0) | 0 (0.0) | 13 (100.0) | 0 (0.0) | 1 (0.2) | 14 (2.1) | 0 (0.0) |
| AOM Dose (mg), n (%) | 100 | 0 (0.0) | 0 (0.0) | 4 (30.8) | 0 (0.0) | 0 (0.0) | 4 (0.6) | 0 (0.0) |
|  | 200 | 0 (0.0) | 0 (0.0) | 3 (23.1) | 9 (24.3) | 0 (0.0) | 12 (1.8) | 0 (0.0) |
|  | 300 | 0 (0.0) | 0 (0.0) | 3 (23.1) | 15 (40.5) | 44 (7.8) | 62 (9.4) | 0 (0.0) |
|  | 400 | 0 (0.0) | 0 (0.0) | 3 (23.1) | 13 (35.1) | 517 (92.2) | 533 (80.4) | 251 (100.0) |
|  | Oral only | 29 (100.0) | 23 (100.0) | 0 (0.0) | 0 (0.0) | 0 (0.0) | 52 (7.8) | 0 (0.0) |
| CYP Inhibitor, n (%) | None | 17 (58.6) | 5 (21.7) | 13 (100.0) | 37 (100.0) | 521 (92.9) | 593 (89.4) | 250 (99.6) |
|  | CYP2D6 strong | 12 (41.4) | 0 (0.0) | 0 (0.0) | 0 (0.0) | 0 (0.0) | 12 (1.8) | 0 (0.0) |
|  | CYP3A4 strong | 0 (0.0) | 18 (78.3) | 0 (0.0) | 0 (0.0) | 4 (0.7) | 22 (3.3) | 1 (0.4) |
|  | CYP2D6 weak | 0 (0.0) | 0 (0.0) | 0 (0.0) | 0 (0.0) | 26 (4.6) | 26 (3.9) | 0 (0.0) |
|  | CYP3A4 weak | 0 (0.0) | 0 (0.0) | 0 (0.0) | 0 (0.0) | 6 (1.1) | 6 (0.9) | 0 (0.0) |
|  | CYP2D6 weak and CYP3A4 strong | 0 (0.0) | 0 (0.0) | 0 (0.0) | 0 (0.0) | 2 (0.4) | 2 (0.3) | 0 (0.0) |
|  | CYP2D6 weak and CYP3A4 weak | 0 (0.0) | 0 (0.0) | 0 (0.0) | 0 (0.0) | 1 (0.2) | 1 (0.2) | 0 (0.0) |
|  | CYP2D6 strong and CYP2D6 weak and CYP3A4 weak | 0 (0.0) | 0 (0.0) | 0 (0.0) | 0 (0.0) | 1 (0.2) | 1 (0.2) | 0 (0.0) |
| CYP(2D6) Genotype, n (%) | Extensive metabolizer | 24 (82.8) | 0 (0.0) | 0 (0.0) | 7 (18.9) | 487 (86.8) | 518 (78.1) | 222 (88.4) |
|  | Poor metabolizer | 5 (17.2) | 0 (0.0) | 0 (0.0) | 0 (0.0) | 31 (5.5) | 36 (5.4) | 6 (2.4) |
|  | Unknown | 0 (0.0) | 23 (100.0) | 13 (100.0) | 30 (81.1) | 43 (7.7) | 109 (16.4) | 23 (9.2) |
| Continuous | | | | | | | | |
| Age (y) | Mean (SD) | 30.00 (8.26) | 28.65 (7.99) | 39.54 (12.18) | 44.86 (10.38) | 39.93 (10.65) | 39.37 (10.92) | 41.32 (10.28) |
|  | Median | 28.00 | 27.00 | 41.00 | 47.00 | 40.00 | 40.00 | 42.00 |
|  | Min, Max | 18.0, 44.0 | 18.0, 43.0 | 19.0, 57.0 | 19.0, 61.0 | 18.0, 60.0 | 18.0, 61.0 | 18.0, 60.0 |
| Body Weight (kg) | Mean (SD) | 72.92 (12.47) | 75.88 (9.01) | 84.66 (17.15) | 85.42 (14.50) | 81.08 (21.89) | 80.85 (20.89) | 83.10 (21.05) |
|  | Median | 74.00 | 77.00 | 84.80 | 84.20 | 77.10 | 77.60 | 79.50 |
|  | Min, Max | 45.9, 94.6 | 62.0, 91.0 | 60.8, 109.8 | 52.7, 117.0 | 40.8, 175.0 | 40.8, 175.0 | 47.7, 164.2 |
| AOM, aripiprazole once monthly; CYP, cytochrome P450; DDI, drug-drug interaction; Max, maximum; Min, minimum; n/N, number of subjects; SD, standard deviation.  ^a^ Study 31-07-247 excluding the oral only and 50 mg/25 mg treatment arms. | | | | | | | | |

| Table S3. Summary Statistics of Additional Covariates | | | | | | | | |
| --- | --- | --- | --- | --- | --- | --- | --- | --- |
| Subject Characteristic | Description | Study  31-98-207 (DDI CYP2D6) N = 29 | Study  31-98-206 (DDI CYP3A4) N = 23 | Study  CN138020 (AOM Single Dose) N = 13 | Study  31-05-244 (AOM Multiple Dose) N = 37 | Study  31-07-246 (Phase 3) N = 561 | Overall N = 663 | Study  31-07-247 N = 251^a^ |
| Categorical | | | | | | | | |
| Injection Site, n (%) | None | 29 (100.0) | 23 (100.0) | 0 (0.0) | 0 (0.0) | 0 (0.0) | 52 (7.8) | 0 (0.0) |
|  | Arm | 0 (0.0) | 0 (0.0) | 4 (30.8) | 0 (0.0) | 0 (0.0) | 4 (0.6) | 0 (0.0) |
|  | Thigh | 0 (0.0) | 0 (0.0) | 9 (69.2) | 0 (0.0) | 0 (0.0) | 9 (1.4) | 0 (0.0) |
|  | Gluteus maximus | 0 (0.0) | 0 (0.0) | 0 (0.0) | 37 (100.0) | 561 (100.0) | 598 (90.2) | 251 (100.0) |
| Needle Size, n (%) | Oral only | 29 (100.0) | 23 (100.0) | 0 (0.0) | 0 (0.0) | 545 (97.1) | 597 (90.0) | 0 (0.0) |
|  | 1.5 | 0 (0.0) | 0 (0.0) | 13 (100.0) | 37 (100.0) | 7 (1.2) | 57 (8.6) | 135 (53.8) |
|  | 2 | 0 (0.0) | 0 (0.0) | 0 (0.0) | 0 (0.0) | 9 (1.6) | 9 (1.4) | 116 (46.2) |
| Injection Volume, n (%) | Oral only | 29 (100.0) | 23 (100.0) | 0 (0.0) | 0 (0.0) | 0 (0.0) | 52 (7.8) |  |
|  | 1 | 0 (0.0) | 0 (0.0) | 4 (30.8) | 0 (0.0) | 0 (0.0) | 4 (0.6) | 0 (0.0) |
|  | 1.5 | 0 (0.0) | 0 (0.0) | 0 (0.0) | 15 (40.5) | 44 (7.8) | 59 (8.9) | 0 (0.0) |
|  | 2 | 0 (0.0) | 0 (0.0) | 3 (23.1) | 22 (59.5) | 517 (92.2) | 542 (81.7) | 251 (100.0) |
|  | 3 | 0 (0.0) | 0 (0.0) | 3 (23.1) | 0 (0.0) | 0 (0.0) | 3 (0.5) | 0 (0.0) |
|  | 4 | 0 (0.0) | 0 (0.0) | 3 (23.1) | 0 (0.0) | 0 (0.0) | 3 (0.5) | 0 (0.0) |
| Continuous | | | | | | | | |
| Body Mass Index (kg/m^2^) | Mean (SD) | 23.55 (2.99) | 24.20 (2.47) | 28.02 (4.70) | 28.50 (3.62) | 28.34 (7.21) | 27.99 (6.87) | 28.78 (6.63) |
|  | Median | 23.80 | 23.80 | 30.20 | 27.50 | 27.00 | 26.50 | 27.30 |
|  | Min, Max | 17.1, 29.2 | 19.8, 29.4 | 22.4, 35.4 | 20.2, 34.9 | 15.4, 61.4 | 15.4, 61.4 | 17.8, 50.2 |
| Lean Body Mass (kg) | Mean (SD) | 55.43 (10.05) | 58.75 (7.08) | 60.74 (9.00) | 62.30 (8.17) | 54.94 (10.36) | 55.62 (10.27) | 56.27 (10.24) |
|  | Median | 51.50 | 59.60 | 60.50 | 63.10 | 53.60 | 54.80 | 55.10 |
|  | Min, Max | 37.5, 71.1 | 45.2, 70.6 | 44.4, 77.1 | 41.7, 76.4 | 26.0, 85.3 | 26.0, 85.3 | 35.0, 87.8 |
| Alanine Aminotransferase (U/L) | Mean (SD) | 18.72 (9.04) | 18.26 (5.64) | 21.54 (10.04) | 27.68 (22.35) | 26.19 (21.78) | 25.58 (20.97) | 26.54 (18.20) |
|  | Median | 16.00 | 17.00 | 20.00 | 23.00 | 21.00 | 20.00 | 21.00 |
|  | Min, Max | 10.0, 47.0 | 10.0, 31.0 | 9.0, 38.0 | 9.0, 139.0 | 6.0, 331.0 | 6.0, 331.0 | 4.0, 133.0 |
| Aspartate Aminotransferase (U/L) | Mean (SD) | 23.24 (5.20) | 20.78 (3.27) | 22.77 (10.39) | 22.35 (10.77) | 22.58 (10.28) | 22.54 (9.96) | 23.86 (10.74) |
|  | Median | 23.00 | 21.00 | 20.00 | 20.00 | 21.00 | 21.00 | 21.00 |
|  | Min, Max | 14.0, 37.0 | 16.0, 26.0 | 7.0, 44.0 | 11.0, 71.0 | 8.0, 119.0 | 7.0, 119.0 | 9.0, 82.0 |
| Alkaline Phosphatase (U/L) | Mean (SD) | 60.72 (16.36) | 59.30 (12.24) | 89.38 (40.18) | 80.11 (22.10) | 75.32 (22.56) | 74.67 (22.93) | 76.53 (29.01) |
|  | Median | 60.00 | 59.00 | 71.00 | 78.00 | 74.00 | 72.00 | 72.00 |
|  | Min, Max | 32.0, 98.0 | 42.0, 89.0 | 49.0, 178.0 | 39.0, 141.0 | 22.0, 200.0 | 22.0, 200.0 | 36.0, 307.0 |
| Bilirubin, Total (mg/dL) | Mean (SD) | 0.69 (0.35) | 0.83 (0.38) | 0.42 (0.18) | 0.37 (0.21) | 0.53 (0.30) | 0.53 (0.31) | 0.52 (0.27) |
|  | Median | 0.60 | 0.70 | 0.40 | 0.40 | 0.47 | 0.47 | 0.40 |
|  | Min, Max | 0.3, 1.8 | 0.3, 1.7 | 0.1, 0.8 | 0.1, 1.2 | 0.2, 2.2 | 0.1, 2.2 | 0.2, 1.6 |
| AOM, aripiprazole once monthly; CYP, cytochrome P450; DDI, drug-drug interaction; Max, maximum; Min, minimum; n/N, number of subjects; SD, standard deviation.  ^a^ Study 31-07-247 excluding the oral only and 50 mg/25 mg treatment arms. | | | | | | | | |

| Table S4. Population Pharamcokinetic Model Prior to Addition of Phase 3 AOM Data | | | | |
| --- | --- | --- | --- | --- |
| Parameter | Final Parameter Estimate | | Magnitude of Interindividual Variability (%CV) | |
|  | Population Mean | %SEM | Final Estimate | %SEM |
| K_a_: oral first-order absorption rate (1/h) | 0.540 | 23.5 | 65.88 | 30.4 |
| CL: clearance for EM (L/h) | 3.31 | 6.0 | 39.37 | 16.7 |
| CL: clearance for PM (L/h) | 1.61 | 11.4 |  |  |
| CL: proportional change in CL for CYP2D6 inhibitor | -0.511 | 11.8 |  |  |
| CL: proportional change in CL for CYP3A4 inhibitor | -0.237 | 13.1 |  |  |
| V_c_: central volume (L) | 118 | 24.0 | 87.75 | 41.9 |
| Q_1_: intercompartmental CL (L/h) | 0.591 | 10.7 | NE | NA |
| V_p1_: peripheral volume (L) | 118 | 45.7 | NE | NA |
| Q_2_: second intercompartmental CL (L/h) | 28.8 | 28.2 | NE | NA |
| V_p2_: second peripheral volume (L) | 134 | 11.6 | NE | NA |
| R_1_: rate of oral dose into depot (mg/h) | 9.33 | 16.2 | NE | NA |
| IM K_a_: IM Depot first-order absorption rate (1/h) | 0.00105 | 10.5 | 54.86 | 20.3 |
| Probability of EM | 0.90 | Fixed | NE | NA |
| RV (%CV) | 24.23 | 9.1 | NA | NA |
| Minimum value of the objective function = 13291.832 | | | | |
| AOM, aripiprazole once monthly; CYP, cytochrome P450; EM, extensive metabolizer; IM, intramuscular; NA, not applicable; NE, not estimated; %CV, percent coefficient of variation; %SEM, percent standard error of the mean; PM, poor metabolizer; RV, residual variability | | | | |

**Study References**

| Study | Reference |
| --- | --- |
| 31-98-206 | Hunt T (PPD Pharmaco, Austin, TX). A double-blind, placebo-controlled study of the effects of orally administered ketoconazole on aripiprazole (OPC-14597) pharmacokinetics in healthy adult male and female subjects. Final report Jun 2001. Rockville (MD): Otsuka Maryland Research Institute: 2001. 1154 p. Internal Report No.: 31-98-206. |
| 31-98-207 | Hoelscher D (PPD Pharmaco, Austin, TX). An open-label study of aripiprazole (OPC-14597) pharmacokinetics in healthy adults with poor and extensive metabolizer genotypes for cytochrome P450 2D6, and the effect of co-administrated quinidine on aripiprazole pharmacokinetics. Final report Jun 2001. Rockville (MD): Otsuka Maryland Research Institute: 2001. 1314 p. Internal Report No.: 31-98-207. |
| CN138020 | Reeves RA. Assessment of the in vivo release characteristics and safety of an intramuscular depot formulation of aripiprazole in subjects with schizophrenia or schizoaffective disorder. Princeton (NJ): Bristol-Myers Squibb Pharmaceutical Research Institute; 2006 Dec. 3153 p. Document Control No.: 930018046. Internal Report No.: CN138020. |
| 31-05-244 | Mallikaarjun S, Kane J, McQuade RD, et al. Pharmacokinetics, tolerability and safety of multiple doses of aripiprazole depot formulation once monthly in adult schizophrenia: an open-label, parallel-arm, multiple-dose study. Schizophr Res. 2013;150:281-288. |
| 31-07-246 | Kane JM, Sanchez R, Baker RA. Patient-centered outcomes with aripiprazole once-monthly for maintenance treatment in patients with schizophrenia: results from two multicenter, randomized, double-blind studies. Clin Schizophr Relat Psychoses. Summer 2015;9:79-87. |
| 301-07-247 | Fleischhacker WW, Sanchez R, Perry PP. Aripiprazole once-monthly for treatment of schizophrenia: double-blind, randomised, non-inferiority study. Br J Psychiatry. 2014;205:135-144. |


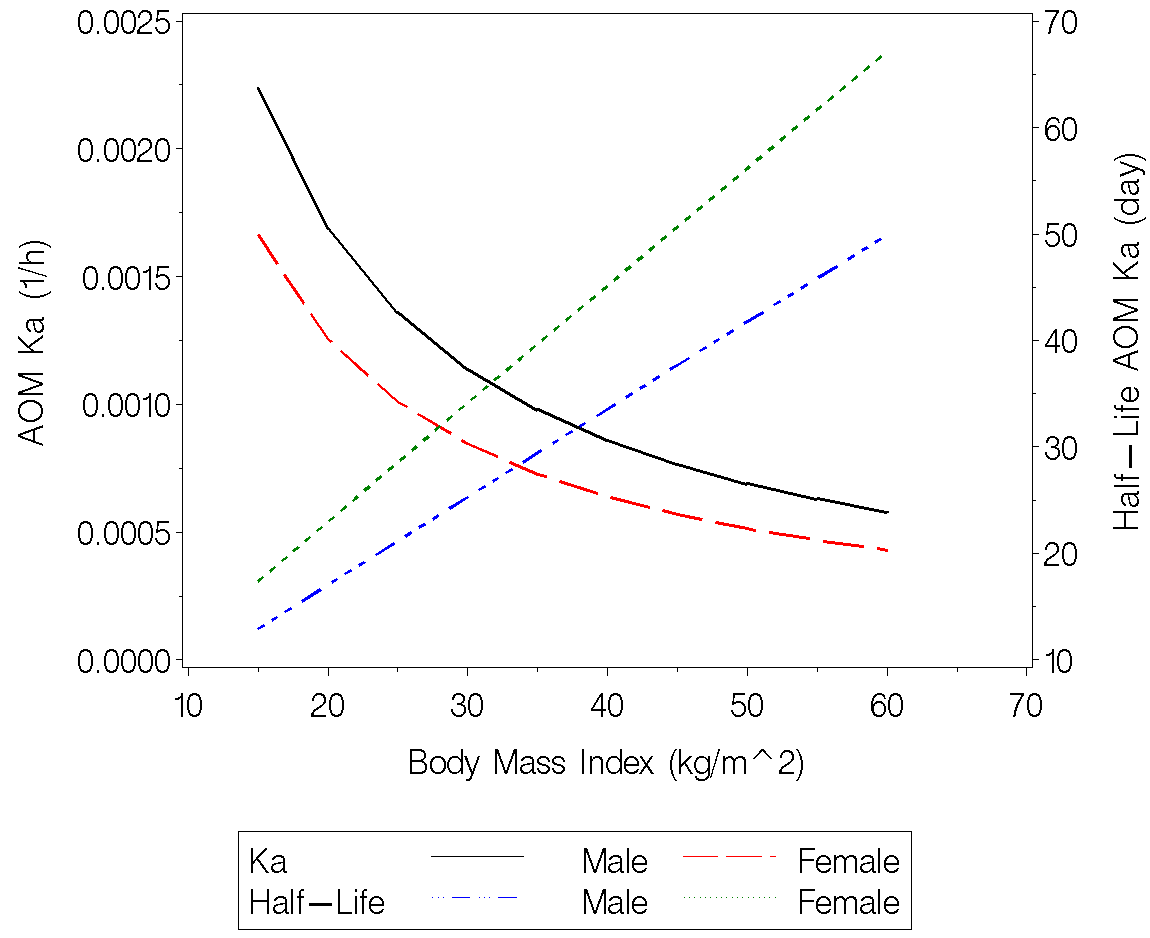


Figure S1. Typical value of AOM K_a_ and half-life of AOM K_a_ versus body mass index stratified by sex.
AOM, aripiprazole once monthly; K_a_, first-order absorption rate constant


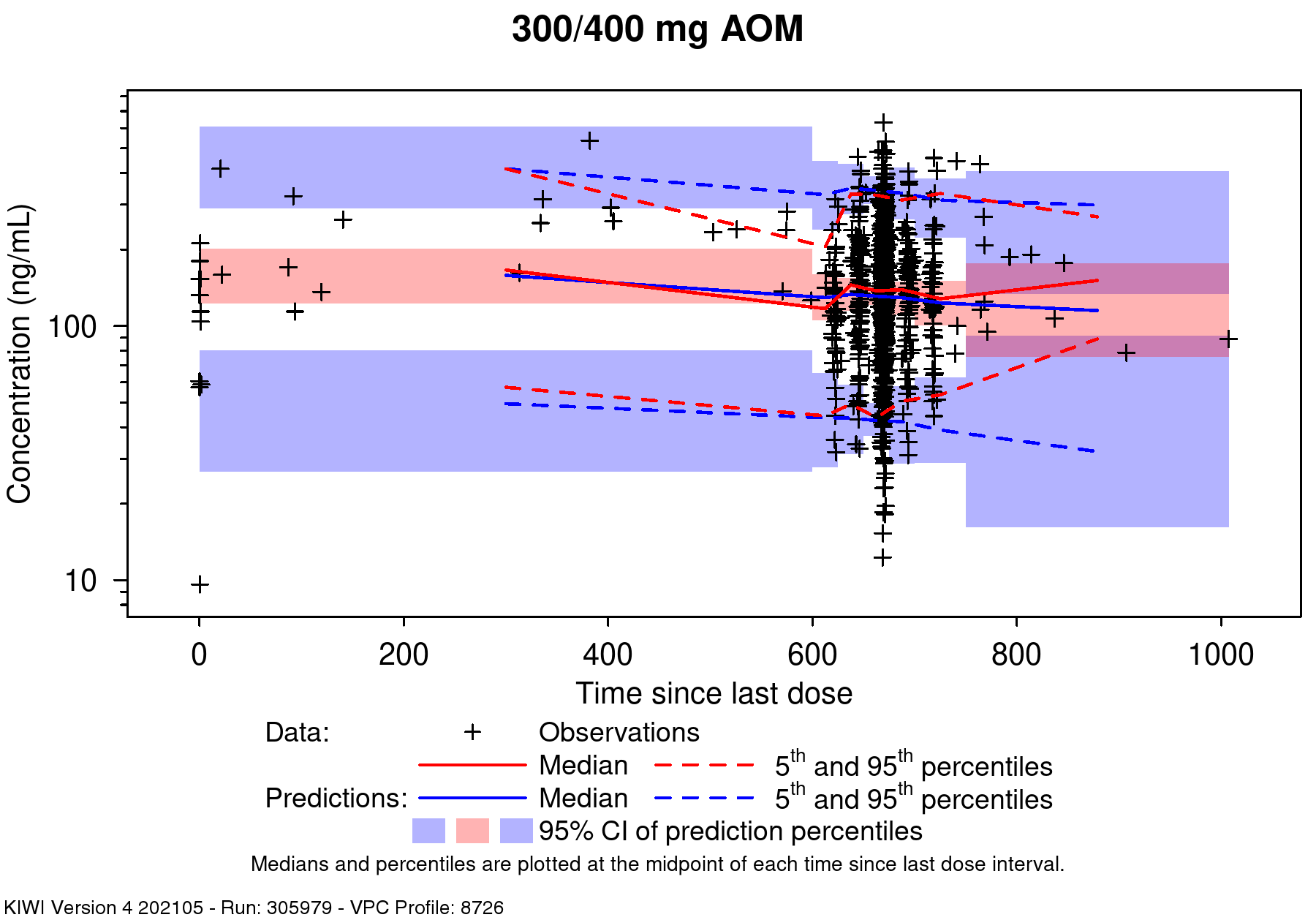


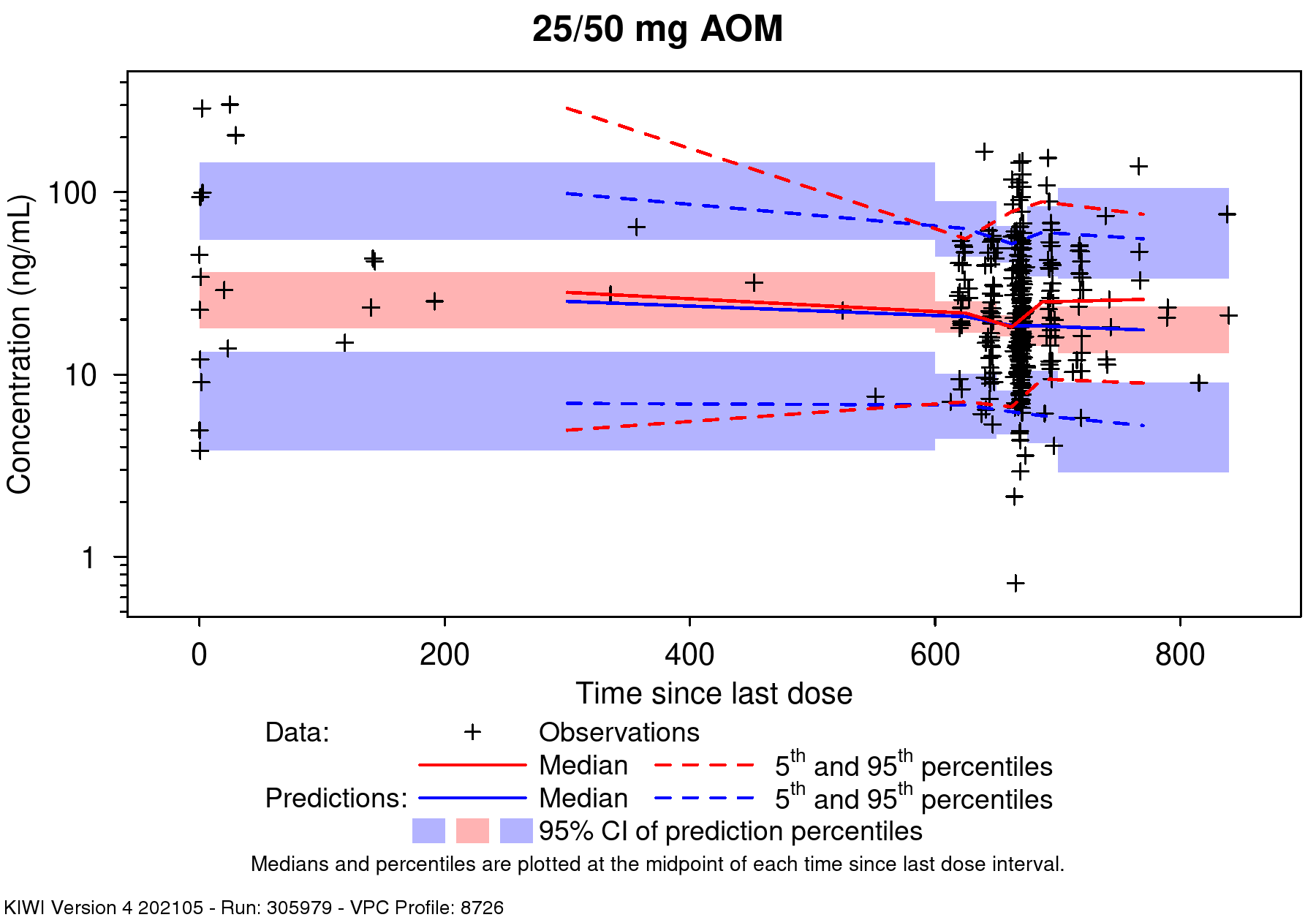


Figure S2. Visual predictive check of the final model applied to the validation dataset, stratified by dose. Top Panel: 400 mg/300 mg. Bottom Panel: 50 mg/25 mg.
AOM, aripiprazole once monthly; CI, confidence interval


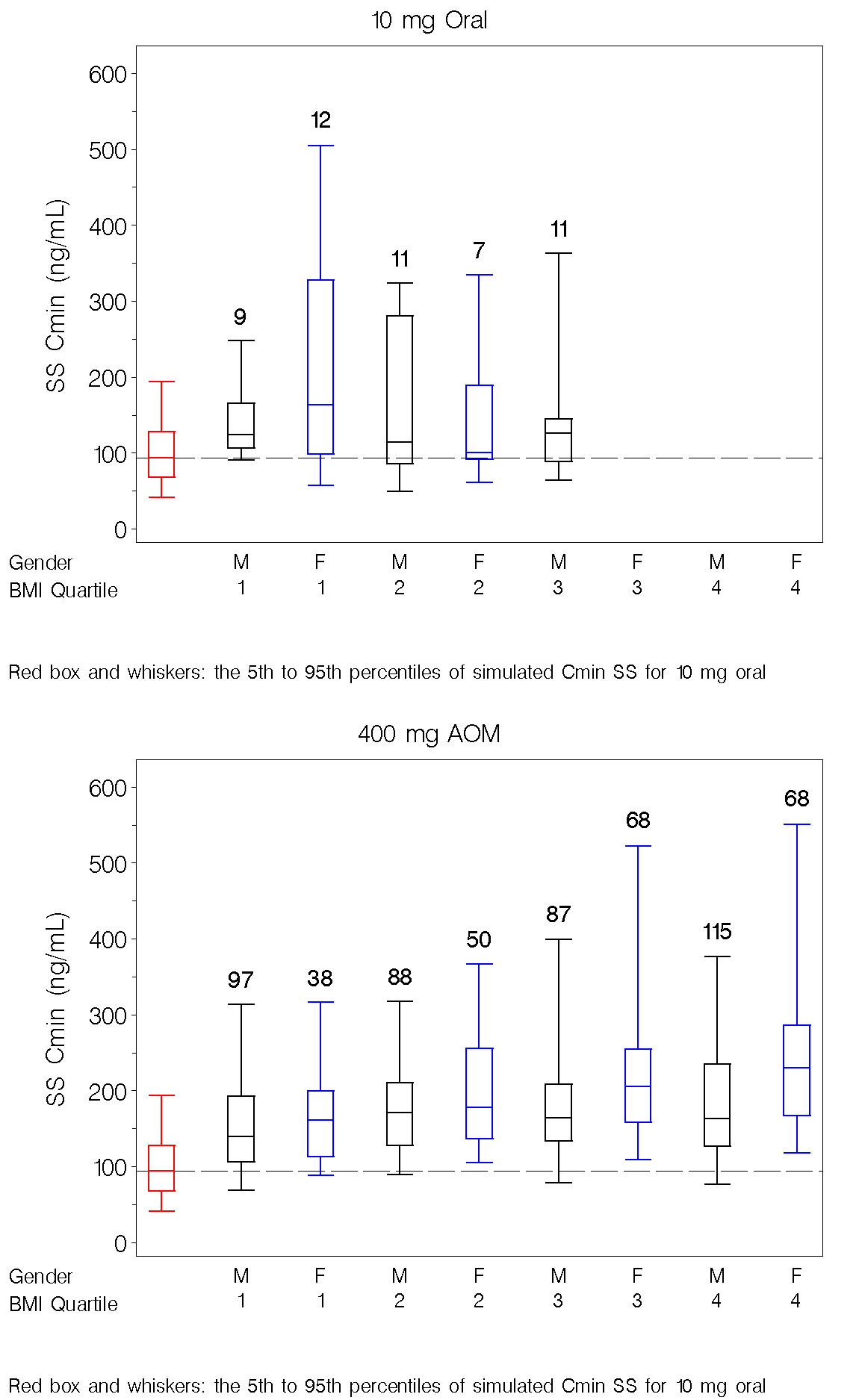

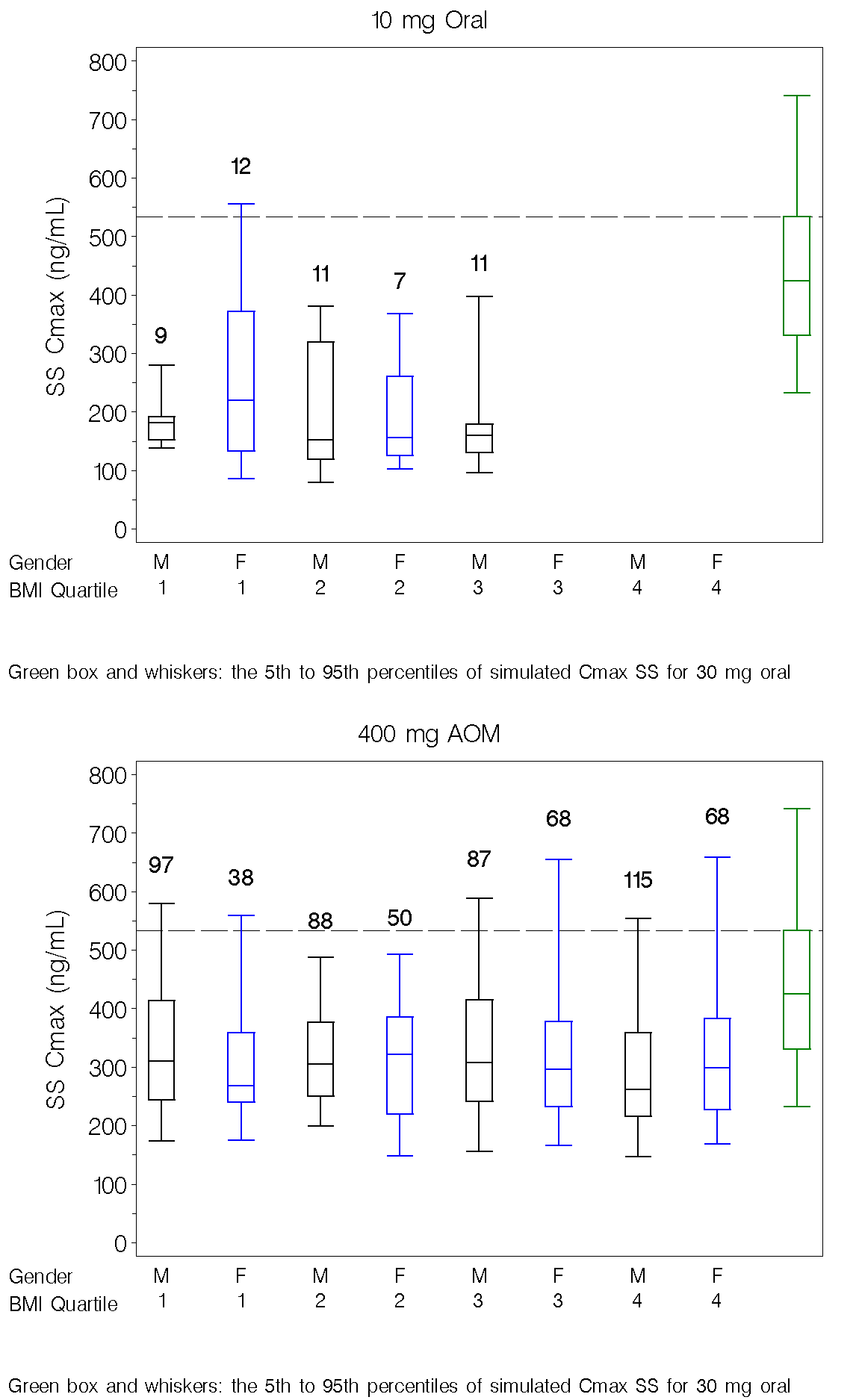


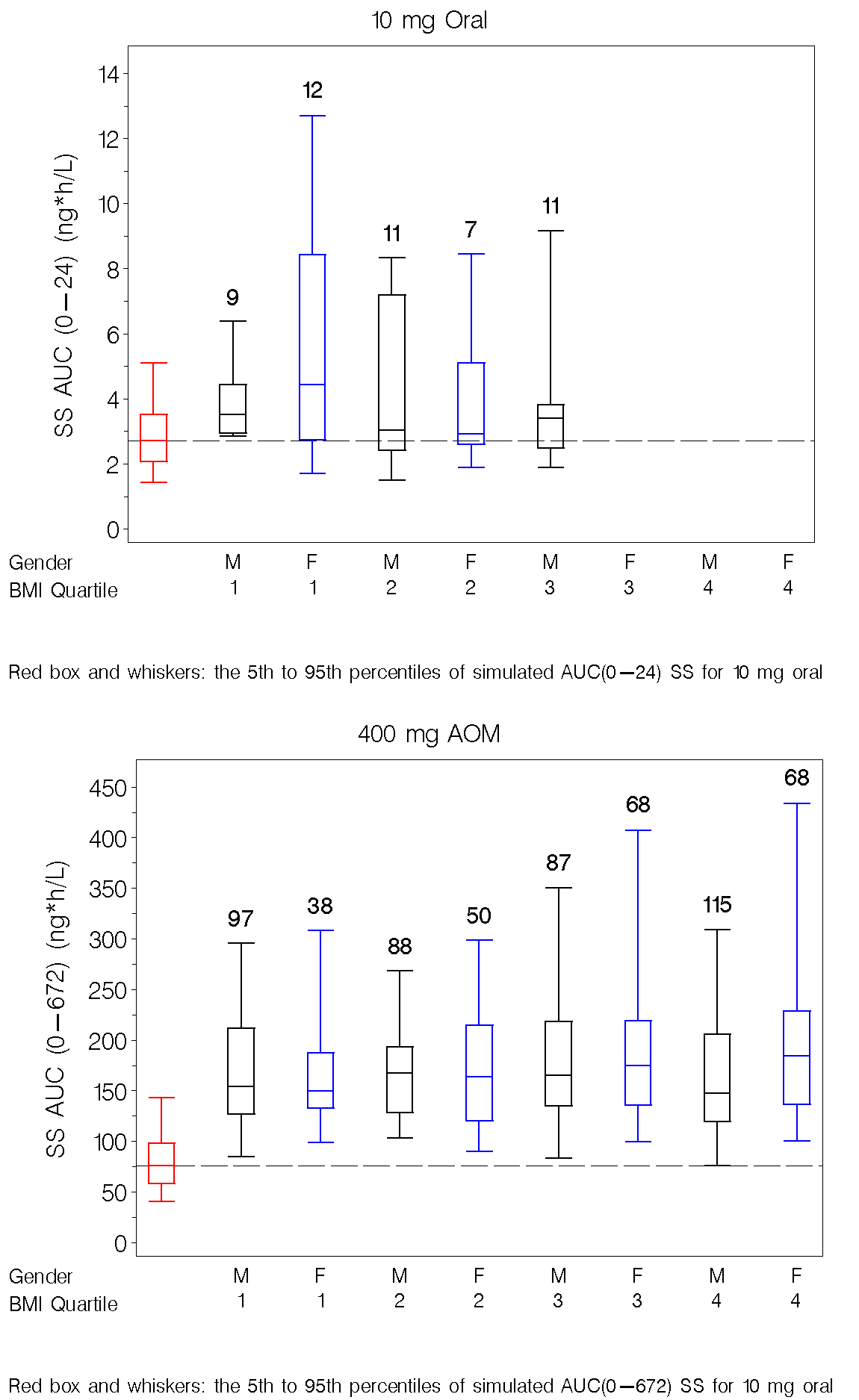


**Male (M) BMI Quartiles:**

1 is BMI ≤ 23.2 kg/m^2^,

2 is 23.2 kg/m^2^ < BMI ≤ 25.7 kg/m^2^,

3 is 25.7 kg/m^2^ < BMI ≤ 29.3 kg/m^2^, and

4 is BMI > 29.3 kg/m^2^.

**Female (F) BMI Quartiles:**

1 is BMI ≤ 22.5 kg/m^2^,

2 is 22.5 kg/m^2^ < BMI ≤ 26.7 kg/m^2^,

3 is 26.7 kg/m^2^ < BMI ≤ 33.9 kg/m^2^, and

4 is BMI > 33.9 kg/m^2^.

Figure S3. Box plots of the model-predicted steady-state C_min_, C_max_, and AUC_0-672_ following 400 mg AOM dosing for subjects in the AOM studies of the model development dataset
AOM, aripiprazole once monthly; AUC_0-672_/AUC (0-672), area under the concentration-time curve from time 0 to 672 hours; BMI, body mass index; C_max_, maximum predicted drug concentration; C_min_, minimum predicted drug concentration; SS, steady state
Note: All boxes represent the 25th, 50th, and 75th percentiles; the whiskers extend to the 5th and 95th percentiles. The unlabeled box to the left (red) represents steady-state dosing of 10 mg oral aripiprazole. The unlabeled box to the right (green) represents steady-state dosing of 30 mg oral aripiprazole. The dashed lines represent the therapeutic window.

| Appendix S1. List of Investigators and Investigational Review Boards, Stratified by Study and Site Number | | | |
| --- | --- | --- | --- |
| Study Number | Site Number | Primary Investigator Clinical Sites | Investigational Review Board |
| 31-98-206 Reference: 1 | 001 | Thomas Hunt, MD, PhD PPD Pharmaco 706A Ben White Blvd., Austin, TX 78704-7016 | Research Consultant’s Review Committee 4009 Banister Lane  One Park Place, Austin, TX 78704 (USA) |
| 31-98-207 Reference: 2 | 001 | David Hoelscher, MD PPD Development 706A Ben White Blvd., Austin, TX 78704-7016 | Research Consultant’s Review Committee 4009 Banister Lane  One Park Place, Austin, TX 78704 (USA) |
| CN138020 Reference: 3 | 001 | Leslie Citrome, MD, MPH Nathan Kline Institute/Rockland Psychiatric Center  (Office & Patient Treatment) Nathan Kline Institute For Psychiatric Research 140 Old Orangeburg Road  Orangeburg, NY 109062 (USA) | Nathan Kline Institute/Rockland Psychiatric Center  Nathan Kline Institute For Psychiatric Research 140 Old Orangeburg Road, Orangeburg, NY 109062 (USA) |
|  | 004 | David Brown, MD Community Clinical Research Inc. (Office & Patient Treatment) Ashwood Retirement and Assisted Living 12151 Hunters Chase, Austin, TX 78729 (USA)  Community Clinical Research Inc. (Patient Treatment) 4411 Medical Parkway Austin, TX 78756 (USA) | Integreview Ethical Review Board 3001 S. Lamar Blvd. Suite 210, Austin, TX 78704 (USA) |
|  | 005 | Mohammed Bari, MD Synergy Clinical Research Center (Office & Patient Treatment) 5577 University Avenue, San Diego, CA 92105 (USA)  University Community Medical Center Laboratory (Patient Treatment) 5550 University Avenue, San Diego, CA 92105 (USA) | Integreview Ethical Review Board 1825 Fortview Road Suite 110, Austin, TX 78704 (USA) |
| 31-05-244 Reference: 4 | 001 | Marina Bussel, MD California Clinical Trials Medical Group 15625 Lakewood Blvd., Paramount, CA 90723 | Sterling Institutional Review Board 6300 Powers Ferry Rd, Suite 600-351  Atlanta, GA 30339 |
|  | 002 | Lev Gertsik, MD California Clinical Trials Medical Group 1560 East Chevy Chase Drive, Suite 140, Glendale, CA 91206 | Sterling Institutional Review Board 6300 Powers Ferry Rd, Suite 600-351  Atlanta, GA 30339 |
|  | 003 | David Walling, PhD Collaborative NeuroScience Network, Inc. 12772 Valley View Street, Suite 3, Garden Grove, CA 92845 | Sterling Institutional Review Board 6300 Powers Ferry Rd, Suite 600-351  Atlanta, GA 30339 |
|  | 004 | Steven Glass, MD CRI Worldwide 1113 Hospital Drive, Suite 202, Willingboro, NJ 08046 | Sterling Institutional Review Board 6300 Powers Ferry Rd, Suite 600-351  Atlanta, GA 30339 |
|  | 006 | Donald Garcia, Jr, MD FutureSearch Trials 4200 Marathon Blvd, Suite 200, Austin, TX 78756 | Sterling Institutional Review Board 6300 Powers Ferry Rd, Suite 600-351  Atlanta, GA 30339 |
|  | 010 | Madeleine Valencerina, MD Clinical Pharmacological Studies, Inc. 10802 College Place, Cerritos, CA 90703 | Sterling Institutional Review Board 6300 Powers Ferry Rd, Suite 600-351  Atlanta, GA 30339 |
|  | 015 | Ricky Mofsen, DO St. Louis Clinical Trials, LC 2639 Miami Street, St. Louis, MO 63118 | Sterling Institutional Review Board 6300 Powers Ferry Rd, Suite 600-351  Atlanta, GA 30339 |
| 31-07-246 Reference: 5 | 001 | Mohammed Bari Synergy Clinical Research Center  1908 Sweetwater Road  National City, CA 91950  United States | Sharon Nelson, MSN, RN, CNS  Schulman Associates Institutional Review Board, Inc.  4290 Glendale-Milford  Cincinnati, OH 45242 United States |
|  | 002 | Arifulla Khan  Northwest Clinical Research Center  1951 152nd Place Northeast  Suite 200  Bellevue, WA 98007  United States | Sharon Nelson, MSN, RN, CNS  Schulman Associates Institutional Review Board, Inc.  4290 Glendale-Milford  Cincinnati, OH 45242 United States |
|  | 003 | Louise M. Thurman  IPS Research Company  1111 North Lee Avenue  Suite 400  Oklahoma City, OK 73103  United States | Sharon Nelson, MSN, RN, CNS  Schulman Associates Institutional Review Board, Inc.  4290 Glendale-Milford  Cincinnati, OH 45242 United States |
|  | 004 | Ronald Brenner  Neurobehavioral Research, Inc.  74 Carman Avenue  Cedarhurst, NY 11516  United States | Sharon Nelson, MSN, RN, CNS  Schulman Associates Institutional Review Board, Inc.  4290 Glendale-Milford  Cincinnati, OH 45242 United States |
|  | 005 | Richard D. Knapp  Florida Clinical Research Center, LLC  2300 Maitland Center Parkway  Suite 230  Maitland, FL 32751  United States | Sharon Nelson, MSN, RN, CNS  Schulman Associates Institutional Review Board, Inc.  4290 Glendale-Milford  Cincinnati, OH 45242 United States |
|  | 006 | Syed Jamal Mustafa  Pacific Institute of Medical Sciences  10634 East Riverside Drive  Suite 100/130  Bothell, WA 98011  United States | Sharon Nelson, MSN, RN, CNS  Schulman Associates Institutional Review Board, Inc.  4290 Glendale-Milford  Cincinnati, OH 45242 United States |
|  | 007 | Mark N. Lerman  Alexian Brothers Center for Psychiatric  Research  1786 Moon Lake Boulevard  Suite 200  Hoffman Estates, IL 60169  United States | Sharon Nelson, MSN, RN, CNS  Schulman Associates Institutional Review Board, Inc.  4290 Glendale-Milford  Cincinnati, OH 45242 United States |
|  | 008 | Anita S. Varma  Research Strategies of Memphis LLC  5395 Estate Office Park Drive  Suite 2  Memphis, TN 38119  United States | Sharon Nelson, MSN, RN, CNS  Schulman Associates Institutional Review Board, Inc.  4290 Glendale-Milford  Cincinnati, OH 45242 United States |
|  | 009 | Naveed Iqbal  Advanced Bio-Behavioral Sciences  Department of Psychiatry  5 West Main Street  Suite 206  Elmsford, NY 10523  United States | Sharon Nelson, MSN, RN, CNS  Schulman Associates Institutional Review Board, Inc.  4290 Glendale-Milford  Cincinnati, OH 45242 United States |
|  | 010 | Randall G. Sullivan  Heartland Pharma Development  116 North Dewey  North Platte, NE 69101  United States | Sharon Nelson, MSN, RN, CNS  Schulman Associates Institutional Review Board, Inc.  4290 Glendale-Milford  Cincinnati, OH 45242 United States |
|  | 011 | Andrew J. Cutler  Florida Clinical Research Center, LLC  2020 26th Ave East  Bradenton, FL 34208  United States | Sharon Nelson, MSN, RN, CNS  Schulman Associates Institutional Review Board, Inc.  4290 Glendale-Milford  Cincinnati, OH 45242 United States |
|  | 012 | Scott D. Segal  Scientific Clinical Research, Inc  c/o Segal Institute for Clinical Research  1065 North East 125th Street  Suite 417  North Miami, FL 33161  United States | Sharon Nelson, MSN, RN, CNS  Schulman Associates Institutional Review Board, Inc.  4290 Glendale-Milford  Cincinnati, OH 45242 United States |
|  | 013 | David W. Brown  Community Clinical Research, Inc  8334 Cross Park Drive  Austin, TX 78754  United States | Sharon Nelson, MSN, RN, CNS  Schulman Associates Institutional Review Board, Inc.  4290 Glendale-Milford  Cincinnati, OH 45242 United States |
|  | 014 | Michael T. Levy  Behavioral Medical Research of Staten Island  500 Seaview Avenue  Suite 200A  Staten Island, NY 10305  United States | Sharon Nelson, MSN, RN, CNS  Schulman Associates Institutional Review Board, Inc.  4290 Glendale-Milford  Cincinnati, OH 45242 United States |
|  | 017 | Rajinder S. Shiwach  InSite Clinical Research  941 York Drive  Suite 205  DeSoto, TX 75115  United States | Sharon Nelson, MSN, RN, CNS  Schulman Associates Institutional Review Board, Inc.  4290 Glendale-Milford  Cincinnati, OH 45242 United States |
|  | 018 | Kashinath G. Yadalam  Lake Charles Clinical Trials  2770 Third Avenue  Suite 340  Lake Charles, LA 70601  United States | Sharon Nelson, MSN, RN, CNS  Schulman Associates Institutional Review Board, Inc.  4290 Glendale-Milford  Cincinnati, OH 45242 United States |
|  | 019 | Zahid Imran  Gulf Coast Research, LLC  7049 Perkins Road  Baton Rouge, LA 70808  United States | Sharon Nelson, MSN, RN, CNS  Schulman Associates Institutional Review Board, Inc.  4290 Glendale-Milford  Cincinnati, OH 45242 United States |
|  | 020 | Martha Edelman  Jamaica Hospital Medical Center  Department of Psychiatry  8900 Van Wyck Expressway  Jamaica, NY 11418  United States | Robert Mendelson, MD and Mahendra Patel, MD  Jamaica Hospital Medical Center Institutional Review  Board/Institutional Privacy Board  8900 Van Wyck Expressway  Jamaica, NY 11418 USA |
|  | 022 | Brian Wise  Colorado Clinical Trials, Inc  c/o Segal Institute for Clinical Research  8671 South Quebec Street  Suite 200  Highlands Ranch, CO 80130  United States | Sharon Nelson, MSN, RN, CNS  Schulman Associates Institutional Review Board, Inc.  4290 Glendale-Milford  Cincinnati, OH 45242 United States |
|  | 024 | Steven L. Dubovsky  University at Buffalo, The State  University of New York  Erie County Medical Center  Department of Psychiatry  462 Grider Street  Buffalo, NY 14215  United States | CoChairs: Steven Dubovsky, MD and Monica Spaulding  MD  Health Sciences IRB  150 Parker Hall  Buffalo, NY 14214 USA |
|  | 026 | Milan K. Joshi  Joshi & Merchant, MD, PA  5500 Knoll North Drive  Suite 290  Columbia, MD 21045  United States | Sharon Nelson, MSN, RN, CNS  Schulman Associates Institutional Review Board, Inc.  4290 Glendale-Milford  Cincinnati, OH 45242 United States |
|  | 029 | Douglas Cosgrove  Belmont Center for Comprehensive Treatment  4200 Monument Road  Philadelphia, PA 19131  United States | Robert Wimmer, MD  Albert Einstein Healthcare Network IRB  5501 Old York Road  Korman Building  Philadelphia, PA 19141 United States |
|  | 032 | Bart A. Sloan  Research Center for Clinical Studies, Inc  71 East Avenue  Suite I  Norwalk, CT 06851  United States | Sharon Nelson, MSN, RN, CNS  Schulman Associates Institutional Review Board, Inc.  4290 Glendale-Milford  Cincinnati, OH 45242 United States |
|  | 033 | Moraima Trujillo  Advanced Research Institute of Miami  3485 West Flagler Street  Suite 300  Miami, FL 33135  United States | Sharon Nelson, MSN, RN, CNS  Schulman Associates Institutional Review Board, Inc.  4290 Glendale-Milford  Cincinnati, OH 45242 United States |
|  | 034 | Lauro AmezcuaPatino  Metropolitan Neuro-Behavioral Institute Research Department  70 North McClintock Suite 4  Chandler, AZ 85226  United States | Sharon Nelson, MSN, RN, CNS  Schulman Associates Institutional Review Board, Inc.  4290 Glendale-Milford  Cincinnati, OH 45242 United States |
|  | 035 | Vijayalakshmi Jayachandran  Confidential Care, Ltd  720 45th Street  Munster, IN 46321  United States | Sharon Nelson, MSN, RN, CNS  Schulman Associates Institutional Review Board, Inc.  4290 Glendale-Milford  Cincinnati, OH 45242 United States |
|  | 036 | Joseph A. Kwentus  Precise Research Centers  3531 Lakeland Drive  Brentwood Plaza Suite 1060  Flowood, MS 39232  United States | Sharon Nelson, MSN, RN, CNS  Schulman Associates Institutional Review Board, Inc.  4290 Glendale-Milford  Cincinnati, OH 45242 United States |
|  | 037 | Wakelin McNeel  Omega Clinical Trials  361 East Whittier Boulevard Suite D  La Habra, CA 90631  United States | Sharon Nelson, MSN, RN, CNS  Schulman Associates Institutional Review Board, Inc.  4290 Glendale-Milford  Cincinnati, OH 45242 United States |
|  | 038 | Vishaal Mehra  Artemis Institute for Clinical Research  8787 Complex Drive Suite 100  San Diego, CA 92123  United States | Sharon Nelson, MSN, RN, CNS  Schulman Associates Institutional Review Board, Inc.  4290 Glendale-Milford  Cincinnati, OH 45242 United States |
|  | 039 | Rick S. Mofsen  St. Louis Clinical Trials, LC  2639 Miami Street  St. Louis, MO 63118  United States | Sharon Nelson, MSN, RN, CNS  Schulman Associates Institutional Review Board, Inc.  4290 Glendale-Milford  Cincinnati, OH 45242 United States |
|  | 040 | Brian S. Bortnick  Comprehensive Neuroscience, Inc  6065 Roswell Road  Suite 820  Atlanta, GA 30328  United States | Sharon Nelson, MSN, RN, CNS  Schulman Associates Institutional Review Board, Inc.  4290 Glendale-Milford  Cincinnati, OH 45242 United States |
|  | 041 | Farrukh Hashmi  Our Lady of Lourdes Hospital at Pasco  DBA, Lourdes Counseling Center  1175 Carondelet Drive  Richland, WA 99354  United States | Sharon Nelson, MSN, RN, CNS  Schulman Associates Institutional Review Board, Inc.  4290 Glendale-Milford  Cincinnati, OH 45242 United States |
|  | 042 | Robert Taylor Segraves  MetroHealth Medical Center  Department of Psychiatry  2500 MetroHealth Drive  Cleveland, OH 44109  United States | David Kuentz, DO  MetroHealth Institutional Review Board  2500 MetroHealth Drive  Cleveland, OH 44109 United States |
|  | 044 | Jeffrey Borenstein  Comprehensive Neuroscience, Inc  The Holliswood Hospital  87-37 Palermo Street  Holliswood, NY 11423  United States | Sharon Nelson, MSN, RN, CNS  Schulman Associates Institutional Review Board, Inc.  4290 Glendale-Milford  Cincinnati, OH 45242 United States |
|  | 045 | Mary L. Stedman  Stedman Clinical Trials, LLC  3212 Cove Bend Drive  Tampa, FL 33613  United States | Sharon Nelson, MSN, RN, CNS  Schulman Associates Institutional Review Board, Inc.  4290 Glendale-Milford Road  Cincinnati, OH 45242 United States |
|  | 046 | Ali A. Kashfi  Ali A. Kashfi, MD, PA  597 Maitland Avenue  Altamonte Springs, FL 32701  United States | Sharon Nelson, MSN, RN, CNS  Schulman Associates Institutional Review Board, Inc.  4290 Glendale-Milford  Cincinnati, OH 45242 United States |
|  | 048 | Juan R. Bustillo  University of New Mexico  Center for Psychiatric Research  1101 Yale NE  Albuquerque, NM 87131  United States | Executive Chair: Mark Holdsworth, PharmD/IRB chairs:  Mark Schuyler, MD and David Doezema, MD  Unviversity of New Mexico  Human Research Review Committee (HRRC) MSC 084560  BMSB Room B71  1 University of New Mexico  Albuquerque, NM 87131 United States |
|  | 049 | Adly Thebaud  Medical Research Group of Central Florida  2725 Rebecca Lane Suite 107  Orange City, FL 32763  United States | Sharon Nelson, MSN, RN, CNS  Schulman Associates Institutional Review Board, Inc.  4290 Glendale-Milford Road  Cincinnati, OH 45242 United States |
|  | 051 | Valentin Isacescu  North County Clinical Research (NCCR)  3230 Waring Court Suite P  Oceanside, CA 92056  United States | Sharon Nelson, MSN, RN, CNS  Schulman Associates Institutional Review Board, Inc.  4290 Glendale-Milford  Cincinnati, OH 45242 United States |
|  | 052 | Daniel F. Chueh  Neuropsychiatric Research Center of Orange County  1440 East 1st Street Suite 406  Santa Ana, CA 92701  United States | Sharon Nelson, MSN, RN, CNS  Schulman Associates Institutional Review Board, Inc.  4290 Glendale-Milford  Cincinnati, OH 45242 United States |
|  | 100 | Todor Tolev  State Psyhiatric Hospital  "Dr. Georgi Kissiov"  First Male Department; First Female Department 1  Magda Petkanova Str,  Radnevo, 6260  Bulgaria | Assoc. Prof. Stefan Denchev, MD Ethics Committee for Multicenter Trials 8, Damian Gruev Str. Sofia, 1303 Bulgaria |
|  | 102 | Valentin Akabaliev  University Multiprofile Hospital for Active Treatment  "Saint George", Psychiatry Clinic  15A, Vassil Aprilov Blvd,  Plovdiv, 4002  Bulgaria | Assoc. Prof. Stefan Denchev, MD Ethics Committee for Multicenter Trials 8, Damian Gruev Str. Sofia, 1303 Bulgaria |
|  | 103 | Georgi Popov  University Multiprofile Hospital for Active Treatment  “Sveta Marina” Varna, Second Psychiatriy Clinic  1, Hristo Smirnenski Str.  Varna, 9010  Bulgaria | Assoc. Prof. Stefan Denchev, MD Ethics Committee for Multicenter Trials 8, Damian Gruev Str. Sofia, 1303 Bulgaria |
|  | 104 | Pepa Dimitrova  University Multiprofile Hospital for Active Treatment  "Dr. Georgi Stranski", Psychiatry Clinic  8A, Georgi Kochev Str.  Pleven, 5800  Bulgaria | Assoc. Prof. Stefan Denchev, MD Ethics Committee for Multicenter Trials 8, Damian Gruev Str. Sofia, 1303 Bulgaria |
|  | 105 | Mitko Mitev  State Psychiatry Hospital Lovech,  Department for Treatment of Depressions, Neuroses, and Border Conditions  1, "Knyaz Aleksander Batenberg"  Lovech, 5500  Bulgaria | Assoc. Prof. Stefan Denchev, MD Ethics Committee for Multicenter Trials 8, Damian Gruev Str. Sofia, 1303 Bulgaria |
|  | 106 | Temenuzhka M Dechkova-Novakova  District Dispensary for Psychiatric Diseases with In-patient Unit,  Male Acute Department; Female Acute Department  20, Tutrakan Blvd.  Rousse, 7000  Bulgaria | Assoc. Prof. Stefan Denchev, MD Ethics Committee for Multicenter Trials 8, Damian Gruev Str. Sofia, 1303 Bulgaria |
|  | 107 | Valentina Genova  State Psychiatry Hospital, Second  Department for Active Treatment of  Severe Psychoses; Third Department for  Additional Treatment; Fourth  Department for Continuous Treatment  Tzerova Koria  Region of Veliko Tarnovo, 5047  Bulgaria | Assoc. Prof. Stefan Denchev, MD Ethics Committee for Multicenter Trials 8, Damian Gruev Str. Sofia, 1303 Bulgaria |
|  | 112 | Irina Ana Dan  Spitalul Clinic de Psihiatrie 'Prof. Al.  Obregia', Sectia 10  Sos. Berceni nr. 10  Bucuresti, 041914  Romania | Prof. Dr. Sava Dumitrescu  National Ethics Committee for the Clinical Study of  Medicine  48 Av. Sanatescu Street  District 1  Bucharest, 011478  Romania |
|  | 113 | Doina Constanta Maria Cozman  Spitalul Clinic Judetean de Urgenta  Cluj, Clinica de Psihiatrie III  Str. Victor Babes nr. 43  Cluj-Napoca, 400012  Romania | Prof. Dr. Sava Dumitrescu  National Ethics Committee for the Clinical Study of  Medicine  48 Av. Sanatescu Street  District 1  Bucharest, 011478  Romania |
|  | 114 | Delia Marina Podea  Spitalul Clinic Judetean de Urgenta  Arad, Sectia Clinica Psihiatrie  Str. Octavian Goga, Nr. 17 Arad, 310022  Romania | Prof. Dr. Sava Dumitrescu  National Ethics Committee for the Clinical Study of  Medicine  48 Av. Sanatescu Street  District 1  Bucharest, 011478  Romania |
|  | 115 | George Mihai Badescu  Spitalul Clinic de Neuropsihiatrie  Craiova, Clinica II Psihiatrie  Calea Bucuresti 149  Craiova, 200620  Romania | Prof. Dr. Sava Dumitrescu  National Ethics Committee for the Clinical Study of  Medicine  48 Av. Sanatescu Street  District 1  Bucharest, 011478  Romania |
|  | 116 | Gheorghe Oros  Spitalul de Neurologie si Psihiatrie  Oradea  Str. Louis Pasteur Nr. 26  Oradea, 410154  Romania | Prof. Dr. Sava Dumitrescu  National Ethics Committee for the Clinical Study of  Medicine  48 Av. Sanatescu Street  District 1  Bucharest, 011478  Romania |
|  | 117 | Gabriel Cristian Marinescu  Spitalul Judetean Arges, Sectia  Psihiatrie  Str. Negru Voda nr. 53  Pitesti, 110069  Romania | Prof. Dr. Sava Dumitrescu  National Ethics Committee for the Clinical Study of  Medicine  48 Av. Sanatescu Street  District 1  Bucharest, 011478  Romania |
|  | 118 | Ana Liana Giurgiuca  Spitalul Clinic de Psihiatrie 'Prof. Al.  Obregia', Sectia 14  Sos. Berceni nr. 10  Bucuresti, 041914 Romania | Prof. Dr. Sava Dumitrescu  National Ethics Committee for the Clinical Study of  Medicine  48 Av. Sanatescu Street  District 1  Bucharest, 011478  Romania |
|  | 119 | Yury A. Suchkov  Nizhny Novgorod Region State  Healthcare Inst "Nizhny Novgorod  Clinical Psych Hosp # 1"  41, Ulianov Street  Nizhny Novgorod, 603155  Russia | Central Ethics Committee: Ramenskaya G.V. Ethical Council of Ministry of Public Health and Social Development of Russian Federation 3 Rakhmanovsky pereulok  Moscow 127994 Russia |
|  | 120 | Boris D. Tsygankov  Educational Institution of Higher  Professional Education "Moscow State Medical and Stomatological University of the Federal Authority for Healthcare and Social Development Regulation" 20/1 Delegatskaya str.  Moscow, 127473  Russia | Central Ethics Committee: Ramenskaya G.V. Ethical Council of Ministry of Public Health and Social Development of Russian Federation 3 Rakhmanovsky pereulok  Moscow 127994 Russia |
|  | 121 | Andrey V. Gribanov  State Healthcare Inst "Lipetsk Regional  Psychoneurological Hospl #1"  Plekhanovo, Gryazinsk District, Lipetsk  Region  Lipetsk, 399083  Russia | Central Ethics Committee: Ramenskaya G.V. Ethical Council of Ministry of Public Health and Social Development of Russian Federation 3 Rakhmanovsky pereulok  Moscow 127994 Russia |
|  | 122 | Natalia I. Buzueva  State Healthcare Inst "Nizhny Novgorod  Regional Psychoneurological Hosp # 1"  12a, Kaschenko Street  Nizhny Novgorod, 603107  Russia | Central Ethics Committee: Ramenskaya G.V. Ethical Council of Ministry of Public Health and Social Development of Russian Federation 3 Rakhmanovsky pereulok  Moscow 127994 Russia |
|  | 124 | Margarita A. Morozova  State Inst "Mental Health Res Cntr of the Russian Academy of Med Sciences"  34, Kashirskoye Shosse  Moscow, 115522  Russia | Central Ethics Committee: Ramenskaya G.V. Ethical Council of Ministry of Public Health and Social Development of Russian Federation 3 Rakhmanovsky pereulok  Moscow 127994 Russia |
|  | 125 | Alexander S. Okhapkin  State Educational Inst of Higher  Professional Education "Smolensk State Mel Academy of the Federal Authority for Healthcare and Social Development  Regulation"  Kirova 46a  Smolensk, 214019  Russia | Central Ethics Committee: Ramenskaya G.V. Ethical Council of Ministry of Public Health and Social Development of Russian Federation 3 Rakhmanovsky pereulok  Moscow 127994 Russia |
|  | 126 | Vladimir A. Tochilov  State Healthcare Inst "St.Nikolas Psych  Hosp"  126, Moyka Embankment  St. Petersburg, 190121  Russia | Central Ethics Committee: Ramenskaya G.V. Ethical Council of Ministry of Public Health and Social Development of Russian Federation 3 Rakhmanovsky pereulok  Moscow 127994 Russia |
|  | 127 | Alexander P. Kotsubinsky  State Inst "St.Petersburg  Psychoneurological Res and Scientific  Inst n.a. V.M. Bekhterev of the Federal  Authority for Healthcare and Social  Development Regulation"  3, Bekhtereva Street  St. Petersburg, 192019  Russia | Central Ethics Committee: Ramenskaya G.V. Ethical Council of Ministry of Public Health and Social Development of Russian Federation 3 Rakhmanovsky pereulok  Moscow 127994 Russia |
|  | 128 | Boris V. Andreyev  St. Petersburg State Healthcare Inst  "Psych Hosp #1 n.a. P.P. Kashenko"  Nikolskoye Village, Gatchina District,  Leningrad Region  St. Petersburg, 188357  Russia | Central Ethics Committee: Ramenskaya G.V. Ethical Council of Ministry of Public Health and Social Development of Russian Federation 3 Rakhmanovsky pereulok  Moscow 127994 Russia |
|  | 130 | Vladimir M. Diligenski  Psychiatry Clinic; KBC DR Dragisa Misovic-Dedinije  Heroja Milana Tepica 1  Belgrade, 11000  Serbia | Vladimir Diligenski  Local Ethics Committee of the Clinical Center “Dr. Dragisa  Misovic-Dedinje”, Psychiatric Clinic, Heroja Milana Tepica  1, Belgrade,  Serbia |
|  | 131 | Gordana Mandic Gajic  Psychiatry Clinic, Military Medical  Academy  Crnotravska 17  Belgrade, 11000  Serbia | Ranko Raicevic  Ethics Committee of Military Medical Academy  Crnotravska 17  Belgrade, 11000  Serbia |
|  | 132 | Goran S. Mihajlovic  Psychiatry Clinic, Clinical Centre  Kragujevac  Zmaj Jovina 30  Kragujevac, 34000  Serbia | Slobodan Jankovic  Ethics Commitee of clinical Centre Kragujevac  Zmaj Jovina 30  Kragujevac, 34000  Serbia |
|  | 135 | Kvetoslav Moravcik  FNsP J. A. Reimana Prešov  Psychiatrické oddelenie I  Holleho 14  Presov, 081 81  Slovakia | MultiCenter Ethics Committee:  Peter Jonas, MD  Etická komisia pri fakultnej nemocnici L.Pasteura Kosice  Rastislavova 43, 04066, Kosice,  Slovenska republika |
|  | 136 | Ivan Doci  Fakultná nemocnica L. Pasteura Košice  II. Psychiatrická klinika  Rastislavova 43  Kosice, 041 90  Slovakia | MultiCenter Ethics Committee:  Peter Jonas, MD  Etická komisia pri fakultnej nemocnici L.Pasteura Kosice  Rastislavova 43, 04066, Kosice,  Slovenska republika |
|  | 137 | Rastislav Korba  Centrum zdravia R.B.K., spol s.r.o.  Psychiatrická ambulancia  MUDr. Pribulu 149  Svidnik, 089 01  Slovakia | MultiCenter Ethics Committee:  Peter Jonas, MD  Etická komisia pri fakultnej nemocnici L.Pasteura Kosice  Rastislavova 43, 04066, Kosice,  Slovenska republika |
|  | 138 | Zuzanna Janikova  Liptovská nemocnica s poliklinikou  Psychiatrické oddelenie  Palúcanská 25  Liptovský Mikuláš, 031 23  Slovakia | MultiCenter Ethics Committee:  Peter Jonas, MD  Etická komisia pri fakultnej nemocnici L.Pasteura Kosice  Rastislavova 43, 04066, Kosice,  Slovenska republika |
|  | 139 | Peter Korcsog  Nemocnice a polikliniky n.o. Bratislava,  Všeobecná nemocnica  Šrobárova 1  Psychiatrické oddelenie  Pracovisko Kraskova 1  Rimavská Sobota, 979 12  Slovakia | MultiCenter Ethics Committee:  Peter Jonas, MD  Etická komisia pri fakultnej nemocnici L.Pasteura Kosice  Rastislavova 43, 04066, Kosice,  Slovenska republika |
|  | 200 | Satheesh Rao  K.S. Hegde Medical Academy  Department of Psychiatry, Centre for  Psychiatric Research  Deralakatte, Karnataka  Mangalore, 575018  India | Prof (Dr) Rajendra Prasad  KSHEMA Ethics Committee  Deralakatta,  Mangalore, Karnataka 575018  India |
|  | 201 | Mahesh Ramanna Gowda  Spandana Nursing Home  Psychiatric  549/46 6th Main 4th Block Rajajinagar  Bangalore, Karnataka 560010 India | Dr Hanamantaray Parappa Karikal  Spandana Ethics Committee  549/46, 6th Main 4th Block  Rajaji Nagar  Bangalore, Karnataka 560010  India |
|  | 202 | Padma Sudhakar Thatikonda  Sri Venkateswara Medical College  Department of Psychiatry, S.V. Medical  College  Andhra Pradesh  Tirupati, 517507  India | Sri. P.Jaya Chandra babu Institutional Ethics Committee, S.V. Medical College Tirupati, Andhra Pradesh 517507 India |
|  | 203 | Ramesh Kumar Mahendru  Mahendru Psychiatric Centre  Psychiatry Department  117/40 Sarvodaya Nagar  Uttar Pradesh  Kanpur, 208005 India | Dr S.N Singh  Mahendru Psychiatric Centre Ethics Committee  117/40 Sarvodaya Nagar  Kanpur, Uttar Pradesh 208005  India |
|  | 204 | Sanjay S. Phadke  Deenanath Mangeshkar Hospital &  Research Centre  Neuropsychiatry Department  Erandwane, Maharashtra  Pune, 411004  India | Dr. Ushatai Khire Institutional Ethics Committee Deenanath Mangeshkar Hospital & Research Centre Erandwane, Maharashtra, Pune, 411004 India |
|  | 206 | Rowena G. Cosca  St. Paul's Hospital  Department of Psychiatry  Room 309, PLC Complex  Iloilo, Western Visayas 5000  Philippines | Fr. Paul Solomia  St. Paul's Hospital  PLC Complex  Iloilo City, 5000  Philippines |
|  | 207 | Ma. Lourdes L. Evangelista  Mariveles Mental Hospital  Medical and Ancillary Department  Poblacion  Mariveles  Bataan, Central Luzon 2105  Philippines | Dr. Marita VT Reyes  National Ethics Committee  Philippine Council for Health & Research Development  3F DOST Building  General Santos Ave, Bicutan,  Taguig City, Metro Manilla  Philippines |
|  | 208 | Efren B. Reyes  National Center for Mental Health  Training and Research Department,  Pavilion 2, Yakal,  Nueve de Pebrero Street  Mandaluyong, NCR 1553  Philippines | Dr. Carmelita Corpuz  National Center for Mental Health Institutional Review  Board / Independent Ethics Committee  9 Nueve de Pebrero St.  Mandaluyong, 1553  Philippines |
|  | 209 | Ponciano Z. Jerez  Veterans Memorial Medical Center  Ward 5, Department of Psychiatry  Veterans Memorial Medical Center  North Avenue  Diliman  Quezon City, NCR 1104  Philippines | Emerita A Barrencehea. MD  VMMC Ethics Committee  Veterans Memorial Medical Centre  North Avenue, Diliman  Quezon City 1100  Philippines |
|  | 210 | Yen-Kuang Yang  National Cheng Kung University Hospital  Department of Psychiatry  138, Shing-Li Rd  Tainan, 704  Taiwan | Ih-Jen Su  Joint Institutional Review Board  3rd Floor, Chi-Ten Building, No. 201, Sec. 2, Shih-Pai Rd.,  Taipei, 112  Taiwan |
|  | 212 | Yuan-Hwa Chou  Taipei Veterans General Hospital  Department of Psychiatry  201, Sec 2. Shih-Pai Road  Taipei, 112  Taiwan | Tung-Ping Su  Institutional Review Board, Taipei Veterans General  Hospital  3F, Chi-Ten Building, No. 201, Sec. 2, Shih-Pai Rd.,  Taipei 112,  Taiwan |
|  | 214 | Shih-Ku Lin  Taipei City Hospital, Songde Branch  309, Song-Der Rd  Taipei, 110  Taiwan | Lin Chi-Hung  Joint Institutional Review Board  3rd Floor, Chi-Ten Building, No. 201, Sec. 2, Shih-Pai Rd.,  Taipei, 112  Taiwan |
|  | 215 | Suarn Singh  Hospital Bahagia Ulu Kinta  Klinik Pakar  Tanjong Rambutan, Perak 31250  Malaysia | Dato'Dr Chang Kian Meng  Medical Research and Ethics Committee (MREC)  Ministry of Health,  National Institute of Health c/o Institute for Health  Management,  Jalan Rumah Sakit, Bangsar, 59000,  Kuala Lumpur  Malaysia |
|  | 216 | Normala Ibrahim  University Putra Malaysia  Porta Kabin, Klinik Pakar UPM  Hospital Kajang  Jalan Semenyih, Kajang  Hulu Langat  Ampang Jaya  Selangor, 43000  Malaysia | Dato'Dr Chang Kian Meng  Medical Research and Ethics Committee (MREC)  Ministry of Health,  National Institute of Health c/o Institute for Health  Management,  Jalan Rumah Sakit, Bangsar, 59000,  Kuala Lumpur  Malaysia |
|  | 217 | Maniam Thambu  Universiti Kebangsaan Malaysia  Medical Centre  Psychiatry Department  Jalan Yaacob Latiff, Bandar Tun Razak  Cheras, Kuala Lumpur 56000  Malaysia | Prof Dr. Rohaizak Muhammad  Jawatankuasa Etika Penyelidikan (Research Ethics  Committee)  Universiti Kebangsaan Malaysia Medical Centre  Level 1, Blok Klinikal, Universiti Kebangsaan Malaysia  Medical Centre,  Jalan Yaakob Latiff,Bandar Tun Razak,  56000 Cheras  Kuala Lumpur,  Malaysia |
|  | 218 | Ahmad Hatim Sulaiman  Universiti Malaya Medical Centre  (UMMC)  Department of Psychological Medicine  Kuala Lumpur, Wilayah Persekutuan  50603  Malaysia | Prof Looi Lai Meng  Medical Ethics Committee, UMMC  5th Floor Manara Timur, Lembah Pantal  University Malaya Medical Centre,  59100 Kuala Lumpur  Malaysia |
|  | 219 | Ramanathan Sathianathan  Madras Medical College & Government  General Hospital  Department of Psychiatry  Chennai, Tamil Nadu 600003 India | Prof S.K Rajan  The Ethics Committee  Madras Medical College & Government General Hospital  Chennai, Tamil Nadu 600003  India |
|  | 220 | Lakshman S. Dutt  Shri Krishna Prasad Psychiatric Nursing  Home & Research Centre  111 Shriji Complex, Opp. Mahakant  Building, Near Ellis Bridge Post Office,  Behind V. S. Hospital  Ahmedabad, Gujarat 380006  India | Dr. G.R Bhagat National Ethics Committee A/301 Chandanbala Appartment, Suvidha Shopping centre,  Paldi Ahmedabad, Gujarat 380007 India |
|  | 306 | Ricardo M. Corral  Fundación para el Estudio y  Tratamiento de las Enfermedades  Mentales  Cerviño 4634 Piso 5to Dpto B  Buenos Aires, C1425AHQ  Argentina | Central Ethics Committee: Dr. Carlos A. Barclay Comité de Etica Independiente en Investigación Clínica "Dr Carlos A Barcaly"  Larrea 1381 3ro A  Buenos Aires, C1117ABK Argentina |
|  | 309 | Carlos A. Morra  Sanatorio "Prof. León S. Morra". S.A.  Av. Sagrada Familia esq Nazaret  Cordoba, Cordoba X5009BIN  Argentina | Central Ethics Committee: Dr. Carlos A. Barclay Comité de Etica Independiente en Investigación Clínica "Dr Carlos A Barcaly"  Larrea 1381 3ro A  Buenos Aires, C1117ABK Argentina |
|  | 310 | Luis D. Mosca  Instituto Nacional de Psicopatología  Campichuelo 215  Buenos Aires, C1405BOA  Argentina | Central Ethics Committee: Dr. Carlos A. Barclay Comité de Etica Independiente en Investigación Clínica "Dr Carlos A Barcaly"  Larrea 1381 3ro A  Buenos Aires, C1117ABK Argentina |
|  | 311 | Gerardo M. García Bonetto  Clinica Privada Integral de Psiquiatria  San Nicolas S.R.L  David Luque 966 B°  Cordoba Capital, Cordoba X5004ALB Argentina | Central Ethics Committee: Dr. Carlos A. Barclay Comité de Etica Independiente en Investigación Clínica "Dr Carlos A Barcaly"  Larrea 1381 3ro A  Buenos Aires, C1117ABK Argentina |
|  | 312 | Roxana B. Galeno  Instituto Neurociencias  Olegario V. Andrade 290  Mendoza, Mendoza M5500HYF  Argentina | Central Ethics Committee: Dr. Carlos A. Barclay Comité de Etica Independiente en Investigación Clínica "Dr Carlos A Barcaly"  Larrea 1381 3ro A  Buenos Aires, C1117ABK Argentina |
|  | 313 | Federico C. Cappelli  CENPIA. Centro de Estudios  Neuropsiquiátricos y Psicológicos  Integral Ambulatorio  Calle 41, N° 515 entre 5 y 6  La Plata, Buenos Aires 1900  Argentina | Central Ethics Committee: Dr. Carlos A. Barclay Comité de Etica Independiente en Investigación Clínica "Dr Carlos A Barcaly"  Larrea 1381 3ro A  Buenos Aires, C1117ABK Argentina |
|  | 315 | Hector F. Lamaison  Clínica Privada Neuropsiquiátrica San  Agustin  Calle 55 N° 763  La Plata, Buenos Aires 1900  Argentina | Central Ethics Committee: Dr. Carlos A. Barclay Comité de Etica Independiente en Investigación Clínica "Dr Carlos A Barcaly"  Larrea 1381 3ro A  Buenos Aires, C1117ABK Argentina |
|  | 317 | Enrique I. Kuper  CENydET S.R.L - Centro  Neurobiológico y de Estrés Traumático  - Biopsychomedical Research Group  Marcelo T. De Alvear 768, 4° Piso  Dept A  Ciudad Autónoma de Bs. As., Buenos  Aires C1058AAJ  Argentina | Central Ethics Committee: Dr. Carlos A. Barclay Comité de Etica Independiente en Investigación Clínica "Dr Carlos A Barcaly"  Larrea 1381 3ro A  Buenos Aires, C1117ABK Argentina |
|  | 318 | Jorge Nazar  Centro de Investigaciones Clínicas - Instituto de Neurociencias, Facultad de  Ciencias Médicas, Universidad  Nacional de Cuyo  Av. Libertador 80, Centro Universitario  Mendoza, Mendoza 5500  Argentina | Central Ethics Committee: Dr. Carlos A. Barclay Comité de Etica Independiente en Investigación Clínica "Dr Carlos A Barcaly"  Larrea 1381 3ro A  Buenos Aires, C1117ABK Argentina |
|  | 321 | María L. Moya Palazuelos  Centro para el Desarrollo de la  Medicina y de Asistencia Especializada S.C.  Blvd. Alfonso G. Calderon 2193-A  Interior 802 y 703. Colonia Desarrollo  Urbano Tres Ríos  Culiacan, Sinaloa 80020  Mexico | Joel Antonio Conde Gutierrez, MD  Comité de Revisión Interna y Ética del Hospital Ángeles de  Culiacán  Boulevard Alfonso G Calderon 2193, Colonia Desarrollo  UrbanoTres Ríos  Culicán, Sinaloa 80020  Mexico |
|  | 322 | Felipe J. Ortega Zarzosa  Unidad de Investigación Clínica en  Psiquiatria del Hospital Lomas de San  Luis, Internacional  Av. Palmira 600, Colonia Villas del Pedregal, Torre Médica 5to piso, consultorio 507  San Luis Potosí, San Luis Potosí 78218 Mexico | Alejandro Quesada Sanchez, MD  Comité de Etica Médica, Investigación, Capacitación y  Enseñanza del Hospital de San Juan Luis Internacional  Av. Palmira #600,Colonia Villas del Pedregal  San Luis Potosí, San Luis Potosí, CP 78218  Mexico |
|  | 323 | Juan B. Corral García  Instituto Mexicano de Investigación Clinica, S.A. de C.V.  Durango 216, Colonia Roma  Mexico, DF 6700  Mexico | Pedro Antonio Reyes Lopez, MD  Comité Bioético para la Investigación Clínica S.C  Puebla 422 despacho4, Col Roma Sur  Mexico, DF 06700  Mexico |
|  | 324 | Ricardo Chapa Vazquez  Consultorio del Dr. Ricardo Chapa  Vazquez  Capitán Aguilar 205, Colonia Deportivo  Obispado  Monterrey, Nuevo León 64040  Mexico | Maria de Lourdes Quiroga Ruiz  Comité de Ética del Centro Avanzado de salud Animica  Rio Verde 212, Col. Miravalle  Monterrey, Nuevo Leon, CP 64660  Mexico |
|  | 325 | Sergio J Villaseñor Bayardo  Hospital Civil de Guadalajara "Fray  Antonio Alcalde"  Calle Hospital 278 - Colonia El Retiro  Guadalajara, Jalisco 44280  Mexico | Hector Raul Perez Gomez, MD  Comité de Enseñanza, Investigación y Étia Hospital Civil de  Guadalajara "Fray Antonio Alcalde"  Calle Hospital #278, Colonia el Retiro  Guadalajara, Jalisco, CP 44260  Mexico |
| 31-07-247 Reference: 6 | 400 | David P. Walling, PhD  Collaborative Neuroscience Network, Inc  12772 Valley View, Suite 3  Street  Garden Grove, CA 92845  US | Sharon Nelson, MSN, RN, CNS  Central IRB:  Schulman Associates IRB, Inc.  4445 Lake Forest Drive  Suite 300  Cincinnati, Ohio 45242 |
|  | 401 | Mohammed Y. Alam, MD  American Medical Research,  Inc  1200 Harger Road, Suite 415  Oak Brook, IL 60523  US | Sharon Nelson, MSN, RN, CNS  Central IRB:  Schulman Associates IRB, Inc.  4445 Lake Forest Drive  Suite 300  Cincinnati, Ohio 45242 |
|  | 403 | Amal Chakraburtty, MD  Red River Medical Research Center, LLC  5100 North Brookline Ave, Suite 525  Oklahoma City, OK 73112  US | Sharon Nelson, MSN, RN, CNS  Central IRB:  Schulman Associates IRB, Inc.  4445 Lake Forest Drive  Suite 300  Cincinnati, Ohio 45242 |
|  | 404 | Mark Hamner, MD  Ralph H. Johnson  VA Medical Center  Mental Health Department  109 Bee Street (116)  Charleston, SC 29401  US | David N. Lewin, MD  Local IRB:  Medical University of South Carolina  Institutional Review Board for Human Research  19 Hagood Avenue, Suite 601  Charleston, SC 29425-8570 |
|  | 405 | Michael G. Plopper, MD  Sharp Mesa Vista Hospital  Clinical Research Department  7850 Vista Hill Avenue  San Diego, CA 92123  US | David Bodkin, MD  Local IRB:  Sharp Institutional Review Board  8695 Spectrum Center Boulevard  San Diego, CA 92123 |
|  | 406 | Carlos A. Santana, MD  University of South Florida  Department of Psychiatry and Behavioral Medicine  3515 East Fletcher Avenue  Tampa, FL 33613  US | Theodore D. Schultz, J.D  Local IRB:  Western Institutional Review Board  3535 Seventh Avenue Southwest  Olympia, WA 98502-5010 |
|  | 408 | Gerald A. Maguire, MD  University of California Irvine Medical Center  Department of Psychiatry  101 The City Drive, Building 3  Orange, CA 92868  US | Kenneth G. Linden, MD  Local IRB:  University of California Irvine  IRB  300 University Tower  Irvine, CA 92697 |
|  | 410 | Eduardo Cifuentes, MD  Carolina Clinical Trials, Inc  c/o Segal Institute for Clinical Research  1483 Tobias Gadson Blvd, Suite 107  Charleston, SC 29407  US | Sharon Nelson, MSN, RN, CNS  Central IRB:  Schulman Associates IRB, Inc.  4445 Lake Forest Drive  Suite 300  Cincinnati, Ohio 45242 |
|  | 411 | Adam F. Lowy, MD  Comprehensive Neuroscience, Inc.  Psychiatric Institute of Washington  4228 Wisconsin Avenue, NW  Washington, DC 20016  US | Sharon Nelson, MSN, RN, CNS  Central IRB:  Schulman Associates IRB, Inc.  4445 Lake Forest Drive  Suite 300  Cincinnati, Ohio 45242 |
|  | 412 | Harold H. Harsch, MD  The Medical College of  Wisconsin, Inc. - Psychiatry  Tosa Center, Third Floor  1155 North Mayfair Road  Milwaukee, WI 53226  US | Linda Blust, MD  Local IRB:  The Medical College of Wisconsin, Inc.  Human Research Protection Program  8701 Watertown Plank Road  Milwaukee, WI 53226 |
|  | 413 | Ira D. Glick, MD  Stanford University  Psychiatry and Behavioral Sciences  401 Quarry Road, Room 2122  Stanford, CA 94305  US | David Spiegel, MD  Local IRB:  Administrative Panel on Human Subjects Research  Stanford University  1215 Welch Road, Modular A  Stanford, CA 98305 |
|  | 414 | William Bobo, MD  Vanderbilt Psychiatric  Hospital  Vanderbilt University Medical  Center  1601 23rd Avenue South  Nashville, TN 37212  US | Steven L. Goudy, MD  Local IRB:  Vanderbilt Institutional Review  Board  1313 21st Avenue South  504 Oxford House  Nashville, TN 37232 |
|  | 415 | Rakesh Ranjan, MD  Rakesh Ranjan, MD &  Associates, Inc  12395 McCracken Rd, Suite E  Garfield Heights, OH 44125  US | Sharon Nelson, MSN, RN, CNS  Central IRB:  Schulman Associates IRB, Inc.  4445 Lake Forest Drive  Suite 300  Cincinnati, Ohio 45242 |
|  | 416 | Alberto R. Yataco, MD  IRC Clinics, Inc  (formerly known as  International Research Center)  7801 York Road, Suite 309  Towson, MD 21204  US | Sharon Nelson, MSN, RN, CNS  Central IRB:  Schulman Associates IRB, Inc.  4445 Lake Forest Drive  Suite 300  Cincinnati, Ohio 45242 |
|  | 417 | Teresa Pigott, MD  University of Florida  Department of Psychiatry  Shands Vista  4101 NW 89th Boulevard  Gainesville, FL 32606  US | Theodore D. Schultz, J.D  Local IRB:  Western Institutional Review  Board  3535 Seventh Avenue Southwest  Olympia, WA 98502-5010 |
|  | 418 | Steven G. Potkin, MD  University of California Irvine  Medical Center  Psychiatry & Human Behavior  Building 3, Room 313  101 The City Drive South  Orange, CA 92868-3298  US | Kenneth G. Linden, MD  Local IRB:  University of California Irvine  IRB  5171 California Avenue, Suite  150  Irvine, CA 92697 |
|  | 419 | Philip G. Janicak, MD  Rush University Medical  Center Department of  Psychiatry  2150 West Harrison Street,  Suite 253  Chicago, IL 60612  US | Allen Korenblit, MD  Local IRB:  Rush University Medical Center  Research and Clinical Trials  Administration  Institutional Review Board  707 South Wood Street  Chicago, IL 60612 |
|  | 420 | Tran K. Tran-Johnson, PharmD, PsyD  California Neuropsychopharmacology  Clinical Research Institute,  LLC (CNRI-San Diego, LLC)  446 26th Street, 6th Floor  San Diego, CA 92102  US | Sharon Nelson, MSN, RN, CNS  Central IRB:  Schulman Associates IRB, Inc.  4445 Lake Forest Drive  Suite 300  Cincinnati, Ohio 45242 |
|  | 422 | David Feifel, MD, PhD  University of California San  Diego (UCSD)  Neuropsychiatry and  Behavioral Medicine  200 West Arbor Drive  MC8620 San Diego, CA  92103  US | Michael Caligiuri, Ph.D  Local IRB:  University of California San  Diego (UCSD)  Human Research Protection  Program  La Jolla Village Professional  Center, Suite A208  La Jolla, CA 92093 |
|  | 424 | Donald J. Garcia Jr., MD  FutureSearch Trials  5508 Parkcrest Drive, Suite  300  Austin, TX 78731  US | Sharon Nelson, MSN, RN, CNS  Central IRB:  Schulman Associates IRB, Inc.  4445 Lake Forest Drive  Suite 300  Cincinnati, Ohio 45242 |
|  | 425 | James T. Barker II, MD  New Hope Clinical Research 1447A Highland Avenue, NE  Hickory, NC 28601  US | Sharon Nelson, MSN, RN, CNS  Central IRB:  Schulman Associates IRB, Inc.  4445 Lake Forest Drive  Suite 300  Cincinnati, Ohio 45242 |
|  | 426 | Barbara A. Burtner, MD  Accurate Clinical Trials, Inc  206 Park Place Boulevard  Kissimmee, FL 34741  US | Sharon Nelson, MSN, RN, CNS  Central IRB:  Schulman Associates IRB, Inc.  4445 Lake Forest Drive  Suite 300  Cincinnati, Ohio 45242 |
|  | 427 | Mark Antonio Hernandez, MD  Berma Research Group  4101 NW 4th Street, Suite 208  Plantation, FL 33317  US | Sharon Nelson, MSN, RN, CNS  Central IRB:  Schulman Associates IRB, Inc.  4445 Lake Forest Drive  Suite 300  Cincinnati, Ohio 45242 |
|  | 429 | Kettlie J. Daniels, MD  Kettlie Joseph Daniels MD, Inc  Department of Research  980 South Byrne Road  Toledo, OH 43609  US | Sharon Nelson, MSN, RN, CNS  Central IRB:  Schulman Associates IRB, Inc.  4445 Lake Forest Drive  Suite 300  Cincinnati, Ohio 45242 |
|  | 430 | Morteza Marandi, MD  Comprehensive NeuroScience  11080 East Artesia Boulevard,  Suite A  Cerritos, CA 90703  US | Sharon Nelson, MSN, RN, CNS  Central IRB:  Schulman Associates IRB, Inc.  4445 Lake Forest Drive  Suite 300  Cincinnati, Ohio 45242 |
|  | 431 | Prakash K. Bhatia, MD, PhD  Synergy Escondido  710 East Grand Avenue  Escondido, CA 92025  US | Sharon Nelson, MSN, RN, CNS  Central IRB:  Schulman Associates IRB, Inc.  4445 Lake Forest Drive  Suite 300  Cincinnati, Ohio 45242 |
|  | 432 | Prakash G. Ettigi, MD  Alliance Research Group,  LLC  3212 Cutshaw Avenue, Suite  210  Richmond, VA 23230  US | Sharon Nelson, MSN, RN, CNS  Central IRB:  Schulman Associates IRB, Inc.  4445 Lake Forest Drive  Suite 300  Cincinnati, Ohio 45242 |
|  | 433 | Igor Galynker, MD, PhD  Beth Israel Medical Center  Department of Psychiatry  First Avenue at 16th Street  New York, NY 10003  US | Arnold Winston, MD  Local IRB:  Beth Israel Medical Center IRB  160 Water Street  Floor 24  New York, NY 10038 |
|  | 434 | Jean-Pierre Lindenmayer, MD  Manhattan Psychiatric Center  Psychopharmacology  Research Center  Dunlap 15  Ward’s Island Complex  New York, NY 10035  US | Abel Lajtha  Local IRB:  Nathan Kline Institutional  Review Board  140 Old Orangeburg Road  Building 35  Orangeburg, NY 10962 |
|  | 435 | Rajinder Shiwach, MD  InSite Clinical Research  941 York Drive, Suite 205  DeSoto, TX 75115  US | Sharon Nelson, MSN, RN, CNS  Central IRB:  Schulman Associates IRB, Inc.  4445 Lake Forest Drive  Suite 300  Cincinnati, Ohio 45242 |
|  | 437 | Carlos M. Figueroa, MD  Pasadena Research Institute  1118 East Green Street  Pasadena, CA 91106  US | Sharon Nelson, MSN, RN, CNS  Central IRB:  Schulman Associates IRB, Inc.  4445 Lake Forest Drive  Suite 300  Cincinnati, Ohio 45242 |
|  | 438 | Norman Moore, MD  East Tennessee State  University  Quilien College of Medicine  Department of Psychiatry  100 Central Receiving Drive  Box 70567  Johnson City, TN 37614-1707  US | George Youngberg, MD  Local IRB:  East Tennessee State University/  VA Medical Campus IRB  100 Central Receiving Drive  Box 70565  Johnson City, TN 37614-1771 |
|  | 439 | Terrance J. Bellnier, RPh, MPA, FASCP  Geriatric Pharmacotherapy  Institute, Inc. (GPI)  36 Forest Meadow Trail  Rochester, NY 14624  US | James Cronmiller  Local IRB:  Clinical Investigation  Committee  1425 Portland Avenue  Rochester, NY 14621 |
|  | 441 | Jelena Kunovac, MD  Excell Research  3998 Vista Way, Suite 100  Oceanside, CA 92056  US | Sharon Nelson, MSN, RN, CNS  Central IRB:  Schulman Associates IRB, Inc.  4445 Lake Forest Drive  Suite 300  Cincinnati, Ohio 45242 |
|  | 442 | Armen K. Goenjian, MD  Collaborative Neuroscience  Network, Inc  19401 South Vermont Avenue, Suite F-100  Torrance, CA 90502  US | Sharon Nelson, MSN, RN, CNS  Central IRB:  Schulman Associates IRB, Inc.  4445 Lake Forest Drive  Suite 300  Cincinnati, Ohio 45242 |
|  | 443 | Lee Stevens, MD  Louisiana Clinical Research,  LLC  8730 Youree Drive, Suite B  Shreveport, LA 71115  US | Sharon Nelson, MSN, RN, CNS  Central IRB:  Schulman Associates IRB, Inc.  4445 Lake Forest Drive  Suite 300  Cincinnati, Ohio 45242 |
|  | 444 | Leigh Anne Nelson, PharmD, BCPP  UMKC School of Pharmacy  Division of Pharmacy Practice  Health Sciences Building  2464 Charlotte Street  Kansas City, MO 64108  US | Sommi Roger, Pharm.D  Local IRB:  University of Missouri - Kansas  City  Adult Health Sciences IRB  5319 Rockhill Road  Kansas City, Missouri 64110 |
|  | 445 | C. Lindsay DeVane, PharmD  Medical University of South  Carolina  Department of Psychiatry and  Behavioral Sciences  67 President Street  Charleston, SC 29425  US | David N. Lewin, MD  Local IRB:  Medical University of South  Carolina Office of Research  Integrity  Harborview Office Tower  19 Hagood Avenue  Suite 601 MSC 857  Charleston, SC 29425 |
|  | 449 | Michel A. WoodburyFariña, MD  Michel A. Woodbury-Fariña,  MD  307 Eleanor Roosevelt Street  San Juan, PR 00918 | Sharon Nelson, MSN, RN, CNS  Central IRB:  Schulman Associates IRB, Inc.  4445 Lake Forest Drive  Suite 300  Cincinnati, Ohio 45242 |
|  | 450 | Raymond A. Manning, MD  CNRI - Los Angeles, LLC  8309 Telegraph Road  Pico Rivera, CA 90660  US | Sharon Nelson, MSN, RN, CNS  Central IRB:  Schulman Associates IRB, Inc.  4445 Lake Forest Drive  Suite 300  Cincinnati, Ohio 45242 |
|  | 500 | W. Wolfgang Fleischhacker, MD  Medical University Innsbruck  Anichstr. 35, A-6020  Innsbruck  Austria | Central Ethics Committee:  Univ. Prof. Dr. Ernst Singer  Ethikkomission der Med.  Universitat Wien und des AKH  der Stadt Wien  Borsohkegasse 8b/E06  (Diensizimmergebaude, BT 68)  A-1090, Wien, Austria |
|  | 504 | Chris Bervoets, MD  Psychiatrisch Ziekenhuis Onze-Lieve-Vrouw  Koning Albert I-laan 8  Brugge, 8200  Belgium | Central Ethics Committee:  Prof. Dr. P.P. De Deyn  Comission voor Medische  Ethiek  ZNA - Campus Middelheim  Lindendreef 1  2020 Antwerpen  Belgium |
|  | 505 | Loris Sayan, MD  Mental Health Centre, Prof. Dr Ivan Temkov - Bourgas  EOOD Complex Lazur, Park  Ezero, 8000 Bourgas  Bulgaria | Central Ethics Committee:  Dr. Anastas Stoykov  Ethics Committee for  Multicentre Trials  5 Sveta Nedelya Square  Sofia 1000, Bulgaria |
|  | 506 | Vihra Milanova, MD  UMHAT “Alexandrovska”  1, Georgi Sofiiski Str  Sofia 1431  Bulgaria | Central Ethics Committee:  Dr. Anastas Stoykov  Ethics Committee for  Multicentre Trials  5 Sveta Nedelya Square  Sofia 1000, Bulgaria |
|  | 507 | Stefan Todorov, MD  Diagnostic Consultative  Centre “Tchaika”, Psychiatric  Cabinets  2, Nikola Vaptzarov Str  9000 Varna  Bulgaria | Central Ethics Committee:  Dr. Anastas Stoykov  Ethics Committee for  Multicentre Trials  5 Sveta Nedelya Square  Sofia 1000, Bulgaria |
|  | 508 | Ivo Mitrev, MD  University Multiprofile  Hospital for Active Treatment,  St. Georgi  5A, Vassil Aprilov Blvd,  4002 Plovdiv  Bulgaria | Central Ethics Committee:  Dr. Anastas Stoykov  Ethics Committee for  Multicentre Trials  5 Sveta Nedelya Square  Sofia 1000, Bulgaria |
|  | 509 | Svetlozar Georgiev, MD  State Psychiatry Hospital  Pazardjik  28, Bolnichna Str,  4400 Pazardjik  Bulgaria | Central Ethics Committee:  Dr. Anastas Stoykov  Ethics Committee for  Multicentre Trials  5 Sveta Nedelya Square  Sofia 1000, Bulgaria |
|  | 510 | Iliana Dimitrova Pristavova, MD  MHAT of Neurology and  Psychiatry, Sveti Naum EAD  1 Ljuben Russev str  1113 Sofia  Bulgaria | Central Ethics Committee:  Dr. Anastas Stoykov  Ethics Committee for  Multicentre Trials  5 Sveta Nedelya Square  Sofia 1000, Bulgaria |
|  | 511 | Dragica KozaricKovacic, MD  University Hospital Dubrava  Department of Psychiatry  Avenija G. Suska 6  10000 Zagreb  Croatia | Central Ethics Committee:  Prof. Dinko Vitezic, MD, PhD  Središnje etičko povjerenstvo  Ksaverska cesta 4  10 000 Zagreb  Croatia |
|  | 512 | Gordan Makaric, MD  Psychiatric Hospital Vrapce  University Clinic For General  Psychiatry  Bolnicka cesta 32  10 090 Zagreb  Croatia | Central Ethics Committee:  Prof. Dinko Vitezic, MD, PhD  Središnje etičko povjerenstvo  Ksaverska cesta 4  10 000 Zagreb  Croatia |
|  | 514 | Neven Henisberg, MD  Polyclinic Neuron  Salata 12  10000 Zagreb  Croatia | Central Ethics Committee:  Prof. Dinko Vitezic, MD, PhD  Središnje etičko povjerenstvo  Ksaverska cesta 4  10 000 Zagreb  Croatia |
|  | 519 | Piret Taal, MD  Tartu University Clinics  Psychiatry Clinic, Raja 31  Tartu 50417  Estonia | Central Ethics Committee:  Jaak Põlluste  Central Ethics Committee:  Tallinn Medical Research Ethics  Committee  National Institute for Health  Development  Hiiu 42, Tallinn, 11619, Estonia |
|  | 520 | Ann Veiken, MD  Viljandi Hospital, Psychiatry Clinic Jamejala 71024, Pärsti parish  Viljandi County  Estonia | Central Ethics Committee:  Jaak Põlluste  Central Ethics Committee:  Tallinn Medical Research Ethics  Committee  National Institute for Health  Development  Hiiu 42, Tallinn, 11619, Estonia |
|  | 521 | Anu Arold, MD  MARIENTHALI PPK  MUSTAMAE tee 16  Tallinn 10617  Estonia | Central Ethics Committee:  Jaak Põlluste  Central Ethics Committee:  Tallinn Medical Research Ethics  Committee  National Institute for Health  Development  Hiiu 42, Tallinn, 11619, Estonia |
|  | 522 | Joel Gailledreau, MD  Cabinet Médical Ambroise  Paré  3 Place Mendès France  78990 Elancourt  France | Central Ethics Committee:  Pr JM. Reymann  CPP Ouest V - CHU  Pontchaillou  9 Avenue de la Bataille Flandre  Dunkerque  35000 Rennes  France |
|  | 523 | Bruno Millet, MD  Centre Hospitalier Guillaume  Regnier  108 avenue du Général  Leclerc  BP 60321, 35703 Rennes  France | Central Ethics Committee:  Pr JM. Reymann  CPP Ouest V - CHU  Pontchaillou  9 Avenue de la Bataille Flandre  Dunkerque  35000 Rennes  France |
|  | 524 | Jacques Louvrier, MD  Réseau de Santé  Convergences  Association Le Cheval Bleu  29 - 31 Rue Salengro  62160 Bully Les Mines  France | Central Ethics Committee:  Pr JM. Reymann  CPP Ouest V - CHU  Pontchaillou  9 Avenue de la Bataille Flandre  Dunkerque  35000 Rennes  France |
|  | 525 | Eric Esposito, MD  Centre Hospitalier de Saint  Nazaire  Psychiatrie Adulte Presqu'Ile  Guerandaise  Heinlex 57 rue Michel Ange  BP 414 44606 Saint Nazaire  France | Central Ethics Committee:  Pr JM. Reymann  CPP Ouest V - CHU  Pontchaillou  9 Avenue de la Bataille Flandre  Dunkerque  35000 Rennes  France |
|  | 535 | Ireneusz Kaczorowski, MD Gabinet Lekarski  Psychiatryczny Ireneusz Kaczorowski ul. Kotarbinskiego 14 97-400 Belchatow  Poland | Central Ethics Committee:  Prof. Eugeniusz Małafiej  Komisja Bioetyczna przy  Okregowej Izbie Lekarskiej;  ul. Czerwona 3, Lodz, 93005  Poland |
|  | 536 | Piotr Baranowski, MD, PhD  Przychodnia LekarskoPsychologiczna Persona  Baranowski, Mazurek,  Malyszczak Spolka Partnerska Lekarzy  ul. Kleczkowska 29 50-227 Wroclaw  Poland | Central Ethics Committee:  Prof. Eugeniusz Małafiej  Komisja Bioetyczna przy  Okregowej Izbie Lekarskiej;  ul. Czerwona 3, Lodz, 93005  Poland |
|  | 537 | Krzysztof Klinke, MD  Samodzielny Publiczny Szpital Miejski w Sosnowcu, ul. Szpitalna 1  41-219 Sosnowiec Oddzial  Psychiatryczny ul. Zegadlowicza 3  41-200 Sosnowiec  Poland | Central Ethics Committee:  Prof. Eugeniusz Małafiej  Komisja Bioetyczna przy  Okregowej Izbie Lekarskiej;  ul. Czerwona 3, Lodz, 93005  Poland |
|  | 538 | Mariusz Perucki, MD  Niepubliczny Zaklad Opieki Psychiatrycznej MENTIS ul. Krolowej Jadwigi 4 64-100 Leszno  Poland | Central Ethics Committee:  Prof. Eugeniusz Małafiej  Komisja Bioetyczna przy  Okregowej Izbie Lekarskiej;  ul. Czerwona 3, Lodz, 93005  Poland |
|  | 552 | Viktor Hal, MD  Bajai Szent Rokus Korhaz,  Pszichiátriai osztály  6500 Baja, Rókus u.10  Hungary | Central Ethics Committee:  Gabriella Kardos,  Egészségügyi Tudományos  Tanács Klinikofarmakológiai  Etikai Bizottsága,  1051 Budapest, Arany J. u. 6-8,  Hungary |
|  | 553 | Attila Bojtos, MD  Toldy Ferenc Nonprofit Közhasznú Kft. KórházRendelőintézet  Pszichiátriai osztály  Törteli út 1-3, 2700 Cegléd  Hungary | Central Ethics Committee:  Gabriella Kardos,  Egészségügyi Tudományos  Tanács Klinikofarmakológiai  Etikai Bizottsága,  1051 Budapest, Arany J. u. 6-8,  Hungary |
|  | 554 | Gyorgy OstorharicsHorvath, MD  Petz Aladár Megyei Oktató  Kórház  I.sz.Pszichiátriai és  Mentálhigiénés Osztály  9024 Gyor, Zrínyi u.13  Hungary | Central Ethics Committee:  Gabriella Kardos,  Egészségügyi Tudományos  Tanács Klinikofarmakológiai  Etikai Bizottsága,  1051 Budapest, Arany J. u. 6-8,  Hungary |
|  | 556 | Laszlo Csekey, MD  Dr. Kenessey Albert KórházRendelőintézet I. Pszichiátriai osztály Rákóczi út 125-127  2660 Balassagyarmat  Hungary | Central Ethics Committee:  Gabriella Kardos,  Egészségügyi Tudományos  Tanács Klinikofarmakológiai  Etikai Bizottsága,  1051 Budapest, Arany J. u. 6-8,  Hungary |
|  | 573 | Herman Walter Pretorius, MD, PhD  Weskoppies Hospital  Research Unit  Outpatient Building, Room 19  Weskoppies Hospital Ketjen  Street  Pretoria 0001  South Africa | Prof. CW van Staden  University of Pretoria Research  Ethics Committee  Faculty of Health Sciences  Level 2 Rooms 2.33 & 2.24  HW Snyman South Building -  31 Bophello Road, Pretoria  0001, South Africa |
|  | 574 | Daniel Jan Hendrick Niehaus, MD, PhD  Flexivest 14 Research Centre  37 John X Merriman Street  Oakdale, Bellville, 7530  South Africa | Prof JR Snyman  South African Medical  Association Research Ethics  Committee (SAMAREC)  Block F Castle Walk Corporate  Park  Nossob Street  Erasmuskloof Ext 3  Pretoria 0153, South Africa |
|  | 577 | Evelin Eding, MD  North Estonia Medical Centre  Foundation  Sutiste Tee 19  Tallinn 13419  Estonia | Central Ethics Committee:  Jaak Põlluste  Central Ethics Committee:  Tallinn Medical Research Ethics  Committee  National Institute for Health  Development  Hiiu 42, Tallinn, 11619, Estonia |
|  | 579 | Peeter Laane, MD  OU Jaanson & Laane  Puusepa 3  Tartu 50406  Estonia | Central Ethics Committee:  Jaak Põlluste  Central Ethics Committee:  Tallinn Medical Research Ethics  Committee  National Institute for Health  Development  Hiiu 42, Tallinn, 11619, Estonia |
|  | 580 | Juan Paul Schronen, MD  Cape Trial Centre  Tygervalley Healthcare Centre  43 Old Oak Street, Suite 617  Bellville, 7530, Cape Town  South Africa | Prof JR Snyman  South African Medical  Association Research Ethics  Committee (SAMAREC)  Block F Castle Walk Corporate  Park  Nossob Street  Erasmuskloof Ext 3  Pretoria 0153, South Africa |
|  | 581 | Aleksander Araszkewicz, MD  Szpital Uniwersytecki nr 1 im. dr A. Jurasza w Bydgoszczy Katedra i Klinika Psychiatrii ul. Kurpinskiego 19  85-096 Bydgoszcz  Poland | Central Ethics Committee:  Prof. Eugeniusz Małafiej  Komisja Bioetyczna przy  Okregowej Izbie Lekarskiej;  ul. Czerwona 3, Lodz, 93005  Poland |
|  | 582 | Wlodzimierz Chrzanowski, MD  Prywatne Gabinety Lekarskie  “Promedicus” Anna Agnieszka Tomczak ul. Sw Rocha 13/15 lok. 221 15-879 Bialystok  Poland | Central Ethics Committee:  Prof. Eugeniusz Małafiej  Komisja Bioetyczna przy  Okregowej Izbie Lekarskiej;  ul. Czerwona 3, Lodz, 93005  Poland |
|  | 583 | Dariusz Kosior, MD  Oddzial V  Ogolnopsychiatryczny  Samodzielny Publiczny  Psychiatryczny Zaklad Opieki  Zdrowotnej im. Dr Stanislawa  Deresza w Choroszczy  ul. Plac Z. Brodowicza 1  16-070 Choroszcz  Poland | Central Ethics Committee:  Prof. Eugeniusz Małafiej  Komisja Bioetyczna przy  Okregowej Izbie Lekarskiej;  ul. Czerwona 3, Lodz, 93005  Poland |
|  | 584 | Bartosz Loza, MD, PhD  Samodzielny Publiczny  Zaklad Opieki Zdrowotnej  Szpital Kliniczny w  Krakowie, Oddzial Kliniczny  Klinik Psychiatrii Doroslych,  Dzieci i Mlodziezy, Oddzial  Psychiatrii Doroslych  ul. Kopernika 21a  31-501 Krakow  Poland | Central Ethics Committee:  Prof. Eugeniusz Małafiej  Komisja Bioetyczna przy  Okregowej Izbie Lekarskiej;  ul. Czerwona 3, Lodz, 93005  Poland |
|  | 585 | Agata Szulc, MD, PhD  IV Oddzial  Ogolnopsychiatryczny Klinika  Psychiatrii UM w  Bialymstoku Samodzielny  Publiczny Psychiatryczny  Zaklad Opieki Zdrowotnej im.  Dr Stanislawa Deresza w  Choroszczy  ul. Plac Z. Brodowicza 1  16-070 Choroszcz  Poland | Central Ethics Committee:  Prof. Eugeniusz Małafiej  Komisja Bioetyczna przy  Okregowej Izbie Lekarskiej;  ul. Czerwona 3, Lodz, 93005  Poland |
|  | 586 | Maria Rostworowska, MD  Samodzielny Publiczny  Zaklad Opieki Zdrowotnej  Szpital Kliniczny w  Krakowie, Oddzial Kliniczny  Klinik Psychiatrii Doroslych,  Dzieci i Mlodziezy, Oddzial  Psychiatrii Doroslych  ul. Kopernika 21a  31-501 Krakow  Poland | Central Ethics Committee:  Prof. Eugeniusz Małafiej  Komisja Bioetyczna przy  Okregowej Izbie Lekarskiej;  ul. Czerw |
|  | 587 | Mariya G. AleksandrovaStoycheva, MD  UMHAT D-R Georgi Stranski  EAD  Drug Dependence and  Comorbid Psychiatric  Disorders Clinic  Zh-k Storgozia 113  Pleven 5800  Bulgaria | Central Ethics Committee:  Dr. Anastas Stoykov  Ethics Committee for  Multicentre Trials  5 Sveta Nedelya Square  Sofia 1000, Bulgaria |
|  | 588 | Lachezar Hranov, MD  MHAT of Neurology and  Psychiatry ‘Sveti Naum’ EAD  1, Ljuben Russev str  1113 Sofia  Bulgaria | Dr. Anastas Stoykov  Ethics Committee for  Multicentre Trials  5 Sveta Nedelya Square  Sofia 1000, Bulgaria |
|  | 589 | Milena Strashimirova, MD  Multiprofile Hospital for  Active Treatment, "Doverie"  AD Psychiatric Outpatient  Office Ovcha Kupel II  Sofia 1632  Bulgaria | Central Ethics Committee:  Dr. Anastas Stoykov  Ethics Committee for  Multicentre Trials  5 Sveta Nedelya Square  Sofia 1000, Bulgaria |
|  | 607 | Jung Jin Kim, MD, PhD  In Ho Paik, MD, PhD The Catholic University of  Korea Seoul St. Mary's  Hospital  505 Banpo-dong Seocho-gu  Seoul 137-701  South Korea | Local Ethics Committee:  Catholic University Seoul Mary’s  Hospital Institutional Review Board  505, Banpo-dong, Seocho-gu  Seoul, 137-701  Korea |
|  | 608 | Prof. Chul Eung Kim, MD, PhD Inha University Hospital  7-206, 3-Ga, Sinheung-dong Jung-gu  Incheon 400-711  South Korea | Local Ethics Committee:  Inha University Hospital Institutional  Review Board  7-206, 3-Ga, Sinheung-dong, Jung-gu  Incheon, 400-711  Korea |
|  | 609 | Prof. Jin Sang Yoon, MD, PhD Chonnam National University  Hospital  671 Jebongno Donggu Gwangju 501-757  South Korea | Local Ethics Committee:  Chonnam University Hospital Bio-  Medical Research Ethics Review Board  671 Jebongno Donggu  Gwangju. 501-757  Korea |
|  | 610 | Yong Min Ahn, MD, PhD  Prof. Yong Sik Kim, MD, PhD Seoul National University  Hospital 101 Daehang-ro,  Jongno-gu  Seoul 110-744  South Korea | Local Ethics Committee:  Seoul National University Hospital  Institutional Review Board  101 Daehang-ro, Jongno-gu  Seoul, 110-744  Korea |
|  | 612 | Prof. Ik Seung Chee, MD, PhD Chungnam National  University Hospital 33 Munhwa-ro, Jung-gu  Daejeon 301-721  South Korea | Local Ethics Committee:  Chungnam University Hospital  Institutional Review Board  33 Munhwa-ro, Jung-gu  Daejeon,301-721  Korea |
|  | 613 | Prof. Jung-Seo Yi, MD, PhD Kangnam Sacred Heart  Hospital  948-1 Daerim-1dong Youngdeungpo-Gu  Seoul 150-950  South Korea | Local Ethics Committee:  Hallym University Kangnam Sacred  Heart Hospital Institutional Review  Board  #948-1 Daerim-1dong, Yeongdeungpogu  Seoul, 150-950  Korea |
|  | 614 | Prof. Young Hoon Kim, MD, PhD Inje University Busan Paik  Hospital  633-165 Gaegum-dong Busanjin-gu  Busan 614-735  South Korea | Local Ethics Committee:  Inje University Busan Paik Hospital  Institutional Review Board  633-165 Gaegum-dong, Busanjin-gu,  Busan, 614-735  Korea |
|  | 615 | Kittipong Sanichwankul, MD Suanprung Psychiatric  Hospital  131 Chang Loh Road  Muang Chiang Mai 50100  Thailand | The Ethical review Committee for  Research in Human Subjects, Ministry of  Public Health, Thailand,  Building No. 2 Kromkarnpatre, 3rd Flr.,  Tiwanon Road, Muang,  Nontaburi 11000  Thailand |
|  | 616 | Nipatt Karnjanathanalers, MD Department of Psychiatry,  Faculty of Medicine  Chulalongkorn University  King Chulalongkorn  Memorial Hospital  1837 Rama 4 Road  Pathumwan, Bangkok 10330  Thailand | Institutional Review Board Faculty of  Medicine, Chulalongkorn University  3th Floor Anantamahidol Building  King Chulalongkorn Memorial Hospital  1873 Phraram 4 Road  Pathumwan Bangkok  Thailand 10330 |
|  | 618 | Chawanun Charnsil, MD Maharaj Nakorn Chiang Mai  Hospital  Department of Psychiatry  110/392 Intawaroros Road  Muang Chiangmai 50200  Thailand | Research Ethics Committee 2, Faculty of  Medicine, Chiang Mai University  110 Intavaroros Road  Amphoe Muang, Chiang Mai  Thailand 50200 |
|  | 700 | Claudia C. Barrera Renault, MD  Centro de Estudio y  Tratamiento de Enfermedades  Psiquiátricas CETEP  ASOCIADOS  Soria 626, Las Condes,  Santiago 7580307  Chile | Central Ethics Committee:  Dr. Andrés Stuardo Luengo  Comité Ético Científico del  Servicio de Salud Metropolitano  Oriente  Av Salvador #364, Providencia,  7500922, Santiago  Chile |
|  | 701 | Sergio Gloger Kojchen, MD  PSICOMEDICA  Avenida Salvador #149 -  Office 1101, Providencia,  7500710, Santiago  Chile | Central Ethics Committee:  Dr. Andrés Stuardo Luengo  Comité Ético Científico del  Servicio de Salud Metropolitano  Oriente  Av Salvador #364, Providencia,  7500922, Santiago  Chile |
|  | 702 | Guillermo A. Vergara Harris, MD  Hospital el Pino  Avenida Padre Hurtado  #13560 San Bernardo,  8053095, Santiago Chile | Central Ethics Committee:  Q.F. Verónica Rivera Sciaraffia  Comité Ético Científico del  Servicio de Salud Metropolitano  Sur  Av Santa Rosa #3453, San  Miguel, 8900390, Santiago  Chile |
|  | 707 | Pablo A. Arancibia Soto, MD  Centro de Estudios Clínicos  Ltda. CEC  Guardia Vieja #255 Office  609 Providencia, 7510186,  Santiago Chile | Central Ethics Committee:  Dr. Andrés Stuardo Luengo  Comité Ético Científico del  Servicio de Salud Metropolitano  Oriente  Av Salvador #364, Providencia,  7500922, Santiago  Chile |
|  | 708 | Marcela E. Rojas Segura, MD  Hospital Barros Luco Trudeau  Gran Avenida #3204, San  Miguel Santiago, 8900085  Chile | Central Ethics Committee:  Q.F.C. Verónica Rivera  Sciaraffia  Comité Ético Científico del  Servicio de Salud Metropolitano  Sur  Avenida Santa Rosa 3453, San  Miguel, 8900390, Santiago  Chile |
|  | 711 | Walter M. Torres Cáceres,  MD  Hospital Dr. Hernán  Henríquez Aravena, Montt Nº  115, Temuco, 4781151  Chile | Central Ethics Committee:  D. Patricio Valdés G.  Comité de Ética Científico del  Servicio de Salud Araucanía Sur  Andrés Bello #636 Temuco,  4791301  Chile |
|  | 712 | Sonia V. Larach Walters, MD  Clinica Pedro Montt  Lord Cochrane # 779,  Santiago, 8330838  Chile | Central Ethics Committee:  Dr. Andrés Stuardo Luengo  Comité Ético Científico del  Servicio de Salud Metropolitano  Oriente  Av Salvador #364, Providencia,  7500922, Santiago  Chile |
|  | 713 | Federico Fernando Bertrán  Vives, MD  Hospital Base Valdivia  Avenida Simpson #850,  Valdivia, 5090145  Chile | Central Ethics Committee:  Dra. Ginette Grandjean Obando  Comité de Ética de la  Investigación del Servicio de  Salud Valdivia  Vicente Pérez Rosales #560  Edificio Prales, oficina 307  Valdivia, 5110537  Chile |

**Appendix S2. Bioanalytical Assay Methods**

For Studies 31-98-206 and 31-98-207, OPC-14597 (aripiprazole) and its metabolites (OPC‑14857, OPC-3373, DM-1451, and 2,3-DCPP), and internal standard (OPC-14714) were extracted from human plasma samples using solid phase extraction. The method used a linear calibration curve ranging from 1.000 to 250.0 ng/mL for OPC-14597, OPC-14857, and DM‑1451; and 2.500 to 250.0 ng/mL for OPC-3373 and 2,3-DCPP. Using HPLC-MS/MS, separation was achieved using a TosoHaas TSK-GEL ODS-80Tm (4.6 mm × 150 mm, 3 μm) with a 17-minute gradient. The mobile phases used were 1% Acetic Acid in 2 mM Ammonium Acetate as mobile phase A and 1% Acetic Acid in 50/50 (v/v) Acetonitrile/Methanol as mobile phase B. Using turbo ion spray and positive ion detection, the mass spectrometer conditions were as follows: interface temperature = 70˚C, auxiliary gas = 8.000 mL/min, nebulizer gas = 12 PSI, curtain gas = 8 PSI, nebulizer temperature = 350˚C, dwell time = 200 ms, and the pump flows at 1 mL/min. The mass to charge ratio monitored using MRM for each compound was as follows: OPC-14597 (Parent ion: 448.2; Daughter ion: 285.2), OPC-14857 (Parent ion: 446.2; Daughter ion: 285.2), OPC-3373 (Parent ion: 250.0; Daughter ion: 164.1), DM-1451 (Parent ion: 464.1; Daughter ion: 301.2), 2,3-DCPP (Parent ion: 231.1; Daughter ion: 153.1), and IS: OPC-14714 (Parent ion: 458.2; Daughter ion: 295.2). The calibration standard values were within ± 15% and ± 20% for the LLOQ as well as the quality controls.

For Study CN138020, BMS-337039 (aripiprazole), BMS-337044 (OPC-14857), and internal standard (OPC-14714) were extracted from human plasma samples using solid phase extraction with a calibration range from 1.000 to 250.0 ng/mL. This method was weighted at 1/x^2^ with a quadratic regression. These compounds were extracted from human K3EDTA plasma using a protein precipitation extraction. Using LC-API/MS/MS, separation was accomplished via BDS Hypersil C18 (100 × 2 mm) analytical column. The mobile phase used was 0.19% Formic Acid in 55/45 (v/v) Methanol/ 0.01 M Formate buffer. This system set-up called for an isocratic composition with a flow rate of 300 μL/min. The calibration standard values, as well as two thirds of the quality controls were within ± 15% and ± 20% for the LLOQ.

For Study 31-05-244, OPC-14597 and OPC-14857 were quantitated using solid phase extraction using the method described for Studies 31-98-206 and 31-98-207, except the linear calibration curve ranged from 0.500 to 250.0 ng/mL.

For Studies 31-07-246 and 31-07-247, OPC-14597, OPC-14857, and internal standard Aripiprazole-d8 or OPC-14597-d8 were quantitated using liquid-liquid extraction. The calibration curve ranged from 0.500 to 500.0 ng/mL. Using HPLC-MS/MS, separation was achieved using a Thermo Fischer Betasil Silica-100 (50 × 3 mm, 5 μm). The parent and daughter ions monitored were as follows: OPC-14597 (Parent ion: 448.2; Daughter ion: 285.2), OPC‑14857 (Parent ion: 446.2; Daughter ion: 285.2), and OPC-14597-d8 (Parent ion: 456.3; Daughter ion: 293.3).
